# Monitoring residues of pesticides in food in Brazil: A multiscale analysis of the main contaminants, dietary cancer risk estimative and mechanisms associated

**DOI:** 10.3389/fpubh.2023.1130893

**Published:** 2023-02-22

**Authors:** Juliana Maria Bitencourt de Morais Valentim, Tatiane Renata Fagundes, Mariane Okamoto Ferreira, Pâmela Lonardoni Micheletti, Geise Ellen Broto Oliveira, Milena Cremer Souza, Beatriz Geovana Leite Vacario, Janaína Carla da Silva, Thalita Basso Scandolara, Shaiane Carla Gaboardi, Luciano Zanetti Pessoa Candiotto, Juliana Mara Serpeloni, Fábio Rodrigues Ferreira Seiva, Carolina Panis

**Affiliations:** ^1^Department of Pathological Sciences, Universidade Estadual de Londrina (UEL), Londrina, Brazil; ^2^Department of Biological Sciences, Universidade Estadual do Norte do Paraná (UENP), Jacarezinho, Brazil; ^3^Center of Health Sciences, Universidade Estadual do Oeste do Paraná (UNIOESTE), Blumenau, Brazil; ^4^Université de Montréal (UdeM), Montreal, QC, Canada; ^5^Instituto Nacional de Câncer (INCA), Rio de Janeiro, Brazil; ^6^Instituto Federal Catarinense, Blumenau, Brazil

**Keywords:** pesticide, food intake, cancer risk, environmental exposure, Brazil

## Abstract

**Introduction:**

Pesticides pose a risk for cancer development and progression. People are continuously exposed to such substances by several routes, including daily intake of contaminated food and water, especially in countries that are highly pesticide consumers and have very permissive legislation about pesticide contamination as Brazil. This work investigated the relationship among pesticides, food contamination, and dietary cancer risk.

**Methods:**

Analyzed two social reports from the Brazilian Government: the Program for Analysis of Residues of Pesticides in Food (PARA) and The National Program for Control of Waste and Contaminants (PNCRC).

**Results and discussion:**

First, we characterized the main pesticide residues detected over the maximum limits allowed by legislation or those prohibited for use in food samples analyzed across the country. Based on this list, we estimated the dietary cancer risks for some of the selected pesticides. Finally, we searched for data about dietary cancer risks and carcinogenic mechanisms of each pesticide. We also provided a critical analysis concerning the pesticide scenario in Brazil, aiming to discuss the food contamination levels observed from a geographical, political, and public health perspective. Exposures to pesticides in Brazil violate a range of human rights when food and water for human consumption are contaminated.

## Introduction

Pesticides are a large and heterogeneous group of chemicals used primarily to destroy, repel, or mitigate insects, small animals, weeds, and other undesirable organisms. Chemically these substances are categorized as organochlorines, organophosphates, carbamates, pyrethroids, neonicotinoids, and phenylpyrazoles.

Most of them are considered persistent organic pollutants that can accumulate in the ecosystem and remain in the environment for considerable periods due to their lipophilic characteristic and long half-life (1–3).

Once in the environment, such pesticides can reach the human body through the daily ingestion of contaminated food and drinking water. This exposure may harm humans since these substances are associated with disease development. Neurodegenerative disease (4), respiratory pathologies (5), metabolic disorders (6), reproductive dysfunction (7, 8), and cancer (9, 10) has been linked to pesticides.

In countries whose economy is based on agriculture, this contamination poses a public health issue. In this context, Brazil is at the top of the world's biggest pesticide consumers (11) altogether to China and the United States. Agribusiness is one of the essential activities for the Brazilian economy. Expanding Brazil's export share has been one of the main objectives guiding the Ministry of Agriculture, Livestock, and Supply (MAPA) work (12).

Nonetheless, these active ingredients are not restricted to the production of agricultural commodities. They are commonly found in horticulture and fruit growing, as observed from the reports of the Program for the Analysis of Residues of Pesticides in Food (PARA), coordinated by the National Health Surveillance Agency (ANVISA). This monitoring investigates pesticide residues in food, observing their compliance with the Maximum Residue Limits—MRL allowed and the presence of active ingredients not authorized for a particular crop or banned in the country.

PARA is the most extensive study regarding monitoring the presence of pesticides in foods of plant origin in Brazil, as it has national coverage and all sample analyses are carried out by specialized laboratories. The program is essential, considering that from the results, it is possible to assess the scenario of irregularities and health risks in a country that consumes many pesticides. The activities of PARA began in 2001, and the main goal is to evaluate the levels of pesticide residues that reached the consumer's table. Since then, PARA has coordinated jointly with municipal and state health surveillance agencies and state public health laboratories (13).

Therefore, despite the pivotal role of fruits and vegetables in nutrition and preventing chronic diseases, consuming contaminated food may have critical consequences. As conventional food cultivation uses many pesticides, it poses a chronic risk for cancer development, for example, due to its carcinogenic potential and frequent presence over the maximum residual limits. Studies have developed tools to estimate the dietary cancer index that allows evaluation of the impact of acute and chronic consumption of pesticide-contaminated food on cancer risk (14).

Little information on the cancer risk attributable to food intake is available worldwide, and conflicting results have been reported (15–19). Also, more information is needed concerning the food-derived pesticide-attributable risks for large-scale populations, as in Brazil. In the present study, we investigated literature data about the relationship between food and risk and carcinogenic pathways, considering the main pesticides described in the last Brazilian PARA report. Further, we estimated the Pesticide Residue Index (PRSI) and revised the major mechanisms enrolled regarding its impact on cancer.

## Methods

This study aims to comprehend the multiscale relationship between food contamination by pesticides and the cancer risk attributable to its ingestion. Therefore, it comprises three main parts:

1. The analysis of the pesticide food contamination data from the Brazilian PARA Report.
2. The estimative of the dietary cancer risk related to PARA reported food pesticide contamination.
3. A systematic analysis of literature concerning the consequences of this pesticide exposure.

The number of detections of active ingredients reported in PARA and the concentration detected in mg/kg in the vegetable samples were analyzed. From these data, samples that showed some pesticide concentrations were selected, and then the median per crop was applied. The percentage of pesticide residue detection in samples considered satisfactory by the Vegetal PNCRC was consulted in SDA Ordinance No. 448 of November 17, 2021, published in the Official Gazette of the Federal Government (20).

To assess the Pesticide Residue Index (PRSI), which represents the pesticide residues in a single serving, we used the original equation for Theoretical Maximum Daily Intake (TMDI) (Equation 1). Through some minor changes in the TMDI equation, a second equation was generated and applied to food samples to achieve the PRSI. The comparison between both equations showed us the specific foods and pesticides in these samples have higher than recommended pesticide residues.

For the systematic review of literature, data were obtained from studies available in three critical databases (PubMed, Google Scholar, and Web of Science) on pesticide exposure and its correlation with carcinogenesis. We restricted our search to articles published from 2012 to 2022. We used a combination of the following words in the title and abstract: pesticides, cancer, tumor, and carcinogenesis. Four authors reviewed titles, article abstracts to classify eligible articles, and full text if necessary. All of the included pieces were written and published in English. Animal, *in vitro*, cross-sectional, case-control, cohort, and ecological studies were included.

## Results

### Results of monitoring pesticides residues in food in Brazil: PARA report analysis

Aiming to understand the picture of food contamination in Brazil, we evaluated the results from the PARA report. The first cycle of the program comprised the period between 2001 and 2007 and analyzed nine types of products. The data showed that foods such as strawberries, tomato, and lettuce had the highest levels of unsatisfactory samples, reaching ~50% of sampling by culture. From 2008 onwards, the amount of food analyzed increased each year, reaching 36 different products in the cycle from 2017 to 2020, although, so far, only the first cycle of 14 varieties has been published (Table 1).

**Table 1:** Historical overview of the sampling of in natura foods carried out in PARA (2001–2018).

**PARA report, year**	**Number of vegetables analyzed**	**Varieties analyzed**	**Total samples analyzed**
2001/2007	9	Lettuce, banana, potato, carrot, orange, apple, papaya, strawberry and tomato.	7,321
2008	17	Lettuce, banana, potato, carrot, orange, apple, papaya, strawberry, tomato, pineapple, rice, onion, beans, mango, bell pepper, cabbage and grapes.	1,773
2009	20	Lettuce, banana, potato, carrot, orange, apple, papaya, strawberry, tomato, pineapple, rice, onion, beans, mango, bell pepper, cabbage, grapes, kale, beet and cucumber.	3,130
2010	18	Lettuce, potato, carrot, orange, apple, papaya, strawberry, tomato, pineapple, rice, onion, beans, mango, bell pepper, cabbage, kale, beet and cucumber.	2,488
2011/2012	15	Papaya, cucumber, bell pepper, pineapple, zucchini, lettuce, rice, beans, carrots, orange, apple, corn (cornmeal), strawberry, tomato and grape.	4,690
2013/2015	25	Papaya, banana, mango, cucumber, bell pepper, pineapple, zucchini, beet, potato, onion, cabbage, lettuce, cabbage, rice, beans, carrot, guava, orange, apple, wheat (flour), corn (cornmeal), cassava (flour), strawberry, tomato and grape.	12,051
2017/2018, 1st cycle	14	Bell pepper, guava, carrot, tomato, lettuce, grape, beetroot, orange, pineapple, mango, chayote, sweet potato, garlic and rice.	4,616
2019/2020, 2nd cycle	22	Not published	Not published

Sampling carried out between 2010 and 2018, on average, showed that 63% of the food samples contained some pesticide residue, indicating that most of the food consumed in Brazil has traces of active ingredients due to the spraying of these products. Of this percentage, 27%, on average, are considered unsatisfactory due to the risk they pose to human health. Furthermore, most samples are deemed inadequate because detected pesticides were unauthorized for the crop, which endangers farmers directly exposed to these products and food consumers (21).

The most recent report in Brazil about PARA (released in 2019) deals with the first phase of the 2017–2018 cycle. This cycle analyzed 4,616 samples and searched up to 270 active ingredients of pesticides. Residues of 122 different active ingredients were detected in the samples analyzed, resulting in a total of 8,270 detections.

**Figure 1:**
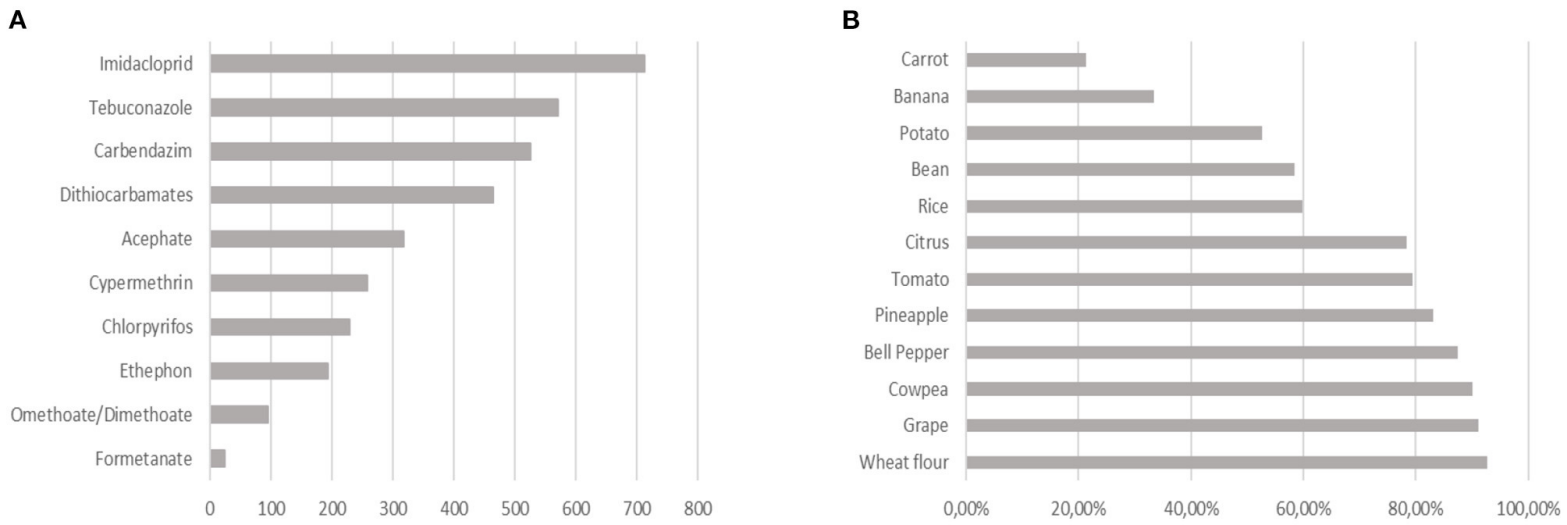
Recognition of pesticide residues according to PARA. **(A)** Main pesticide residues detected in PARA 2017–2018: total detected by active ingredient. **(B)** Percentage of pesticide residue detection about the parameters analyzed in the satisfactory samples of the PNCRC Vegetal 2020.

The most detected pesticides were the insecticide imidacloprid (713 detections) and the fungicides tebuconazole (570 detections) and carbendazim (526 detections) (Figure 1A). Imidacloprid is among the 10 most commercialized pesticides in Brazil (22) and has been associated with the death of bees (23). For this reason, is prohibited in the European Union (24). Carbendazim has been banned in the United States and the European Union for more than a decade, in association with cancer and fetal malformations.

**Figure 2:**
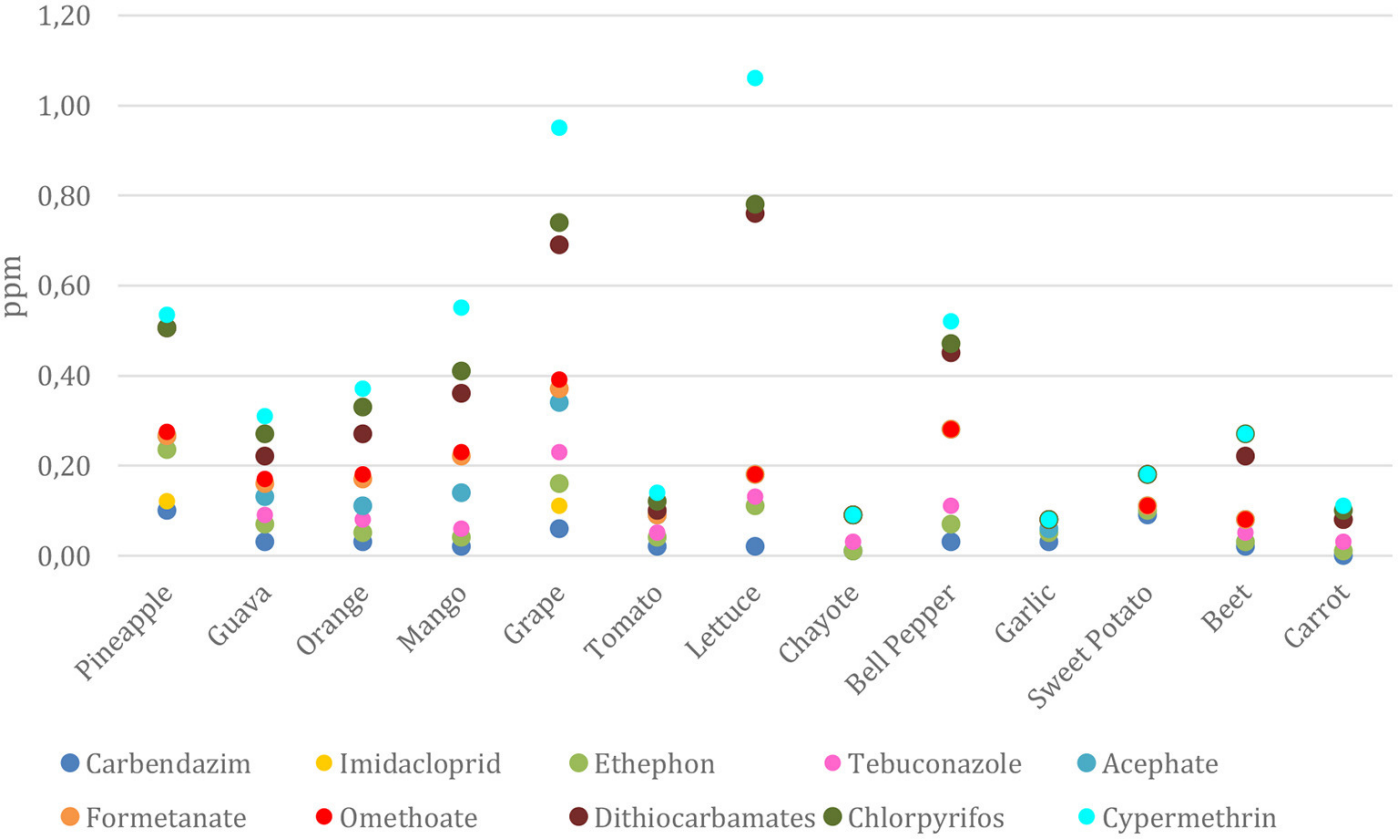
Main pesticide residues detected in PARA 2017–2018: median of values detected per culture (mg/kg).

The foods that presented the highest number of unsatisfactory samples were: peppers (81.9%), guava (42.4%), carrots (39.6%), and tomato (34.8%). Of the total monitored, 41 samples from the 2017–2018 cycle (0.89%) had a potential acute health risk; of this amount, 27 were orange (Figure 2). In addition, 2.9% of the samples, corresponding to 134 units, had 10 or more active ingredients in the same food (13).

Despite the advances, the number of samples analyzed in Brazil seems to be less than ideal, given that throughout PARA (2001–2018), 36,069 samples were analyzed, which represents a little more than a third of what was analyzed in the European Union, only in the year 2018. Another point is that Brazil has been much more permissive about the established MRLs and the pesticides that are used in the national territory, which have been banned for years in European Union countries, as is the case of carbendazim, chlorpyrifos, and acephate, which represents a framework of environmental injustice (25).

Another essential element presented in the reports is the multi-exposure; that is, the consumer, when eating, may be ingesting more than one pesticide at a time. This risk of combined action is not yet estimated in Brazil, but methodologies and pilot studies already exist in the European Union and the United States to guarantee consumer safety (26). This, therefore, is a crucial point for the improvement of PARA.

In addition to PARA, another program has been monitoring pesticide residues in plant samples in Brazil since 2008: the National Program for Control of Waste and Contaminants (PNCRC Vegetal), carried out by the Ministry of Agriculture, Livestock, and Supply (MAPA). The PNCRC/Vegetal has the function of monitoring the quality and safety of products of plant origin produced and consumed throughout the national territory concerning the occurrence of pesticide residues and chemical, physical and biological contaminants. Products of plant origin intended for the domestic and export markets are monitored. MAPA has carried out the PNCRC/Vegetal since 2008, and samples are preferably collected at processing establishments and/or packers, wholesalers, and supply centers. MAPA reports show that on average, 56% of the samples analyzed between 2015 and 2020 had some level of residue (27).

The most recent report, from 2020, shows pesticide residues in 67.17% of the total samples, highlighting the high frequency of residue detection for the following foods: cowpea, grapes, peppers, and wheat flour, which had more than 85% of the samples with the presence of pesticides (Figure 1B). Among the most detected active ingredients are carbendazim, chlorpyrifos, and acephate (20).

### Dietary cancer risk estimative attributable to PARA reported pesticides residues in food

Based on the residues described in Tables 2, 3, we calculated the dietary cancer risk attributable to food contamination. The equation for the Theoretical Maximum Daily Intake (TMDI) (Equation 1) considers the Maximum Residue Limits (MRL) (28) to establish the highest level of pesticides legally tolerated on food or feed crops. For MRL, we only considered the compounds authorized for use in Brazil since the current guidelines of the National Health Surveillance Agency (Anvisa) do not cover unauthorized products. The daily intake of any particular pesticide residue in a given food is obtained by multiplying the residue level in the food (MRL) by the amount consumed (F).

**Table 2:** Pesticide active substances percentages applied over the limit in agricultural crops according with TMDI and PRSI values in 2019.

**Crop**	**Pesticide**	**MLR**	**PR**	**F**	**TMDI**	**PRSI**	**TMDI × PRSI**	**% Over the limit (median)**
Banana	Trifloxystrobin	0.3	0.59	0.13	0.039	0.0767	0.0377	96.6
	Carbendazim	0.5	1.665	0.13	0.065	0.2457	0.1807	278
	Cyazofamid	0.2	1.64	0.12	0.024	0.1968	0.1728	720
	Metalaxyl-M	0.5	0.99	0.12	0.06	0.1188	0.0588	98
	Pyraclostrobin	1	3.5	0.12	0.12	0.42	0.3	250
	Bifenthrin	0.02	0.06	0.086	0.00172	0.00516	0.00344	200
Black bean	Glyphosate	0.05	0.28	0.08	0.004	0.0224	0.0184	460
	Glufosinate	0.05	0.41	0.08	0.004	0.0256	0.0216	540
Papaya	Carbendazim	0.5	1.24	0.16	0.08	0.1984	0.1184	148
	Trifloxystrobin	0.05	0.13	0.16	0.008	0.0208	0.0128	160
Melon	Cypermethrin	0.02	0.04	0.23	0.0012	0.0069	0.0057	275
	Thiamethoxam	0.1	0.145	0.24	0.024	0.0348	0.0108	45
	Carbendazim	0.5	1.6	0.24	0.12	0.384	0.264	220
	Fenpyroximate	0.1	0.3	0.06	0.006	0.018	0.012	200
Soy bean	Glyphosate	10	23.75	0.043	0.43	1.02125	0.59125	137.5
	Cypermethrin	0.05	0.335	0.043	0.00215	0.014405	0.012255	570
Tomato	Bifenthrin	0.02	0.04	0.08	0.0016	0.0032	0.0016	100
	Acephate		0.09254	0.08	0.0016	0.026902	0.0253016	1581.35
	Cyromazine	0.03	0.07	0.08	0.0024	0.0056	0.0032	133.3
Grape	Dimethomorph	2	2.75	0.0992	0.1984	0.2728	0.0744	37.5

**Table 3:** Pesticide active substances percentages applied over the limit in agricultural crops according with TMDI and PRSI values in 2020.

**Crop**	**Pesticide**	**MLR**	**Result**	**C**	**TMDI**	**PRSI**	**TMDI × PRSI**	**% over the limit (median)**
Pinneapple	Carbendazim	0.5	2.078985	0.13	0.065	0.27026805	0.20526805	315.8
Potatoes	Acephate	0.1	0.24916	0.2025	0.02025	0.0504549	0.0302049	149.16
	Imidacloprid	0.05	0.10035	0.2025	0.010125	0.02032088	0.010195875	100.7
	Methamidophos	0.01	0.02216	0.2025	0.002025	0.0044874	0.0024624	121.6
Black bean	Glyphosate	0.05	0.44	0.08	0.004	0.0352	0.0312	780
	Acephate	0.02	0.03	0.08	0.0016	0.0024	0.0008	50
	Glufosinate	0.05	0.18	0.08	0.004	0.018	0.014	350
Cowpea bean	Glyphosate	0.01	0.94	0.048	0.00048	0.05856	0.07152	14900
	AMPA (Glyphosate metabolite)	0.01	0.115	0.048	0.00048	0.00552	0.00528	1100
	Acephate	0.02	0.025	0.048	0.00096	0.0012	0.00024	25
	Glufosinate	0.05	0.33	0.048	0.0024	0.01584	0.01344	560
	Flutriafol	0.2	0.41636	0.06	0.012	0.0249816	0.0129816	108
	Fenpropatrina	0.2	0.280045	0.06	0.012	0.0331209	0.0211209	176
	Chlorfenapyr	0.3	1.55	0.06	0.018	0.093	0.075	416.6
	Cypermethrin	0.02	0.05886	0.06	0.0012	0.0046104	0.0034104	284.2
Tomato	Acephate	0.02	0.13673	0.08	0.0016	0.0109384	0.0093384	583.65
	Bifenthrin	0.02	0.04778	0.08	0.0016	0.0038224	0.0022224	138.9
	Lambda-cyhalothrin	0.05	0.118095	0.08	0.004	0.0094476	0.0054476	136.19
	Cyazofamid	0.5	1.1673	0.0992	0.0496	0.11579616	0.06619616	133.46

Equation 1. Original equation:


$$\begin{array}{l} \text{TMDI}=\text{MRL}\times \text{F} \end{array}$$

TMDI = Theoretical Maximum Daily Intake;

MRL = Maximum Residue Limits (in ppm or mg.kg^−1^); and

F = Recommended food serving size (in mg).

We evaluated both the TMDI and the Pesticide Residue Sample Index (PRSI) for 44 pesticides applied in crops in 2019 and 33 pesticides used in 2020 (Tables 4, 5). PRSI is an adaptation of the original equation (Equation 1) by replacing MRL with accurate pesticide measurements (PR) from all available crops to identify pesticide contamination (PRSI, Equation 2) in food and/or food crops in different Brazilian regions.

**Table 4:** Mechanisms associated with human carcinogenesis following exposure to pesticides.

**Pesticide**	**Type of cancer**	**Associated mechanism**	**Exposure**	**References**
Acephate	Retinoblastoma	–	Prenatal exposure to pesticides in individuals living near application areas.	(29)
Acephate	Testicular germ cell tumors (TGCT)	Endocrine disruptor	Fetal exposure to agricultural endocrine disrupting pesticides.	(30)
Acetamiprid	Liver cancer	–	The presence of acetamiprid in blood samples was detected in the liver cancer group. The blood concentration of a-fetoprotein was higher in both control and cancer groups, showing the risk of developing liver cancer after exposure to acetamiprid.	(31)
Aminomethylphosphonic acid (AMPA)	Breast cancer	–	Exposure to AMPA was evaluated in healthy postmenopausal women and women with breast cancer. The AMPA levels found in the excretion of women with cancer vs. controls were 38% higher.	(32)
Carbendazim	–	–	Possibility of developing cancer after exposure (estimated risk >1) in four areas of Spain (Alzira, Burriana, Benicarló and Benifaió) in babies.	(33)
Carbofuran	Prostate cancer	Men carrying the homozygous wild-type TT genotype at two correlated CDK7 SNPs, rs11744596 and rs2932778, were at increased risk of developing prostate cancer after exposure to carbofuran.	–	(34)
Chlorpyrifos (organophosphate)	Colorectal	–	There was an increased risk of developing cancer and occupational, environmental and food exposure to the insecticide chlorpyrifos.	(35)
Chlorpyrifos	Prostate cancer	–	Men exposed to pesticides and who have the polymorphism in the CYP1A1 enzyme are at greater risk of developing prostate cancer.	(36)
Chlorpyrifos	Breast cancer	–	Women exposed to chlorpyrifos were three times more likely to develop breast cancer when compared to the other pesticides analyzed.	(37)
Chlorpyrifos	Lung cancer	–	Increased risk of developing lung cancer in occupationally exposed individuals.	(38)
Chlorpyrifos	Kidney cancer	–	High risk for the development of renal tumors in occupationally exposed individuals.	(39)
Clothianidin (Neonicotinóides)	Liver cancer	Alters cell growth.	Environmental	(31)
Methyl-Kresoxim		–	Environmental exposure increases the susceptibility to develop astrocytoma.	(40)
Dimethoate	Prostate cancer	–	Increased risk of developing the disease when there is environmental/occupational exposure.	(41)
Dimethoate	Meduloblastoma	–	Higherchances of presenting the disease when mothers were exposed to the environment during pregnancy.	(40)
Fipronil	Bladder cancer	–	Environmental	(42)
Phosmet	Acute lymphoblastic leukemia	–	Pesticide exposure during pregnancy due to residential proximity to agricultural applications may increase childhood ALL risk.	(43)
Glyfosate	Non-Hodgkin lymphoma	–	gbh exposure is associated with increased risk of NHL in humans	(44, 45)
Glyfosate	Acute myeloid leukemia	Users in the highest exposure quartile had an increased risk of acute myeloid leukemia (AML) compared to never users.	Occupational exposure	(46)
Glyfosate	–	B-cell lymphoma was positively associated with phenoxy herbicides and the organophosphate herbicide glyphosate.	Occupational exposure	(47)
Imazalil	Breast cancer	Positive association between dietary exposure and risk of postmenopausal breast cancer was found specifically among overweight and obese women.	Dietary exposure	(18)
Omethoate	–	It can lead to changes in telomere length in workers exposed to the presence of polymorphism in the GSTM1 gene can also influence telomere length.	Occupational exposure	(48)
Omethoate	–	Alteration in p53 and p21 expression levels and may be related to telomere length changes induced by omethoate.	Occupational exposure	(48)
Permethrin	Leukemia	It can cause rearrangements and breaks in genes associated with leukemia in adults and children.	Chronic exposure	(49)
Permethrin	Multiple myeloma	It was observed that there is a high prevalence of its precursor monoclonal gammopathy of undetermined significance, in farmers who use it.	Occupational exposure	(50)
Permethrin	Multiple myeloma	Occupational exposure to Permethrin is associated with an increased risk of developing multiple myeloma.	Occupational exposure	(51)
Permethrin	Leukemia		Mothers who had occupational/daily contact with pesticides during pregnancy may be associated with an increased risk of developing acute leukemia in children occupational exposure.	(52)
Permethrin	–	Decreased telomere length associated with some pesticides including Permethrin.	Occupational exposure	(53)
Permethrin	Lymphoblastic leukemia	Several pesticides have been evaluated for their association with the risk and development of lymphoblastic leukemia in children. there was no association with Permethrin.	Environmental exposure	(54)
Permethrin	Multiple myeloma	Change in hematological parameters in Permethrin applicators.	Occupational exposure	(55)
Permethrin	Non-Hodgkin lymphoma	There was no association between occupational exposure to pyrethroids and non-Hodgkin's lymphoma.	Occupational exposure	(56)
Permethrin	–	Classified pesticides that are potentially carcinogenic by the USEPA and used in large volume.	–	(57)
Propiconazole	Central nervous system tumor	–	A study carried out with mothers who lived in rural areas showed a high risk for medulloblastoma.	(40)
Thiamethoxam	Liver cancer	–	The results showed that exposure through diet increases the chances of liver cancer prevalence.	(31)

**Table 5:** Mechanisms associated with *in vitro* carcinogenesis following exposure to pesticides.

**Pesticide**	**Cell lineage**	**Mechanism**	**References**
Acetamiprid	4T1 breast cancer cells	Acetamiprid induced dose-dependent 4T1 breast cancer cell proliferation, migration, and estrogen receptor interaction.	(58)
Cyfluthrin	H295R human adrenocortical carcinoma cells	Cyfluthrin increased E2 (estradiol) expression	(59)
Cypermethrin	BG-1 ovarian cancer cell	Cypermethrin induced the growth of the ovarian cancer cell line BG-1 and up-regulated cyclin D1 expression.	(60)
Chlorpyrifos	MCF-7 and MDA-MB-231 breast cancer cell lines	Increases cell division by activating the estrogen receptor (ERα).	(61)
Chlorpyrifos	MCF-7 breast cancer cell line	Stimulates angiogenesis progressing to breast cancer	(62)
Chlorpyrifos	Breast cancer cell lines MCF-7 and MDA-MB-231	Increases migration, invasion, phosphorylation	(63)
Chlorpyrifos	A549cell andNCI-H1299 Lung cancer cell	Generates oxidative stress, activates Nrf2 promoting cancer cell survival	(64)
Clothianidin	SH-SY5Y human neuroblastoma cells	Increase cell growth; alters calcium influx; alter gene expression	(65)
Glyfosate	T47D breast cancer cells	Glyphosate promoted the growth of T47D cells *via* estrogen receptors, activation of the ERE (estrogen response element), and, altered estrogen receptors by increasing the expression ratio of ERα and ERβ.	(66)
Glyfosate	MCF-7 and MDA-MB-231 breast cancer cell lines	Low concentration of Roundup dysregulated in both lineages, 11 canonical pathways, the most important being cell cycle repair and DNA damage repair pathways, and alterations in metabolism that can alter mitochondrial oxygen consumption, increase ROS levels, induce hypoxia, cause accumulation of mutations.	(67)
Imidacloprid	Hs578t breast cancer cell lines	Increases CYP19 expression, a key aromatase in estrogen biosynthesis.	(68)
Imazalil	HepG2 cells—human hepatocellular carcinoma	Increased levels of cell proliferation markers, Ki-67 positive nuclei and mcm2 mRNA	(69)
Omethoate	FaDu cell of head and neck cancer	Activation of the Akt/GSK-3β/cyclin D1 pathway, leading to the proliferation of pharyngeal cancer cells.	(70)
Permethrin	K562 cells (chronic myeloid leukemia)	Permethrin induces aneuploidy and structural alterations in the IGH and KMT2A genes, causing fusion of the ETV6-RUNX1 gene in peripheral blood mononuclear cells. It has also been shown to induce fusion of the ETV6-RUNX1 and IGH-BCL2 genes in K562 cells.	(71)
Permethrin	Peripheral blood mononuclear cells	The pesticide at low concentrations induces aberrations in the KMT2A and IGH genes, detected in the interphase and metaphase phases.	(49)
Thiamethoxam	Adenocarcinoma cells (H295R)	Exposure to neonicotinoid pesticides could increase the concentration of the CYP19 enzyme in adenocarcinoma cells (H295R), which cause cell proliferation in breast cancer.	(72)
Thiamethoxam	H295R adrenocortical carcinoma cells	The pesticide induces CYP19 aromatase enzyme activity, increased estradiol and estrone production, CYP3A7 enzyme expression and inhibited estriol in H295R cells.	(73)
Triethanolamine/ trifloxystrobin	SH-SY5Y neuroblastoma cells	It has been observed to cause inhibition of mitochondrial oxidative respiration and to alter the levels of various lipids in neuronal cells.	(74)
Triflumuron	HCT116 Colon Cancer cells	It induces the generation of reactive oxygen species, followed by lipid peroxidation, and an increase in malondialdehyde, it also activates antioxidant enzymes (oxidative stress).	(75)
Triflumuron	HepG2 liver cancer cells	It demonstrated dose-response agonistic activities of HIF-1α at non-cytotoxic concentrations, stimulation of cell migration and invasion.	(76)

4T1, mouse breast cancer cells; A549, human lung adenocarcinoma cells; BG-1, human ovarian cancer cells; FaDu, human hypopharyngeal cancer cells; H295R, human adrenal corticocarcinoma cells; HCT116, human colorectal carcinoma cells; HepG2, human hepatocarcinoma cells; Hs578t, human breast cancer cells; K562, human immortalized myelogenous leukemia cells; MCF-7, human breast cancer cells; MDA-MB-231, human breast cancer cells; NCI-H1299, human lung adenocarcinoma cells; SH-SY5Y, human neuroblastoma cells; T47D, human breast cancer cells.

Equation 2. The equation to identify accurate pesticide contamination:


$$\begin{array}{l} \text{PRSI}=\text{PR}\times \text{F} \end{array}$$

PRSI = Pesticide Residue Sample Index;

PR = Pesticide residues measured in agricultural crops of different Brazilian regions; and

F = Recommended food serving size (in mg), according to the Dietary Guidelines for the Brazilian population (77).

Upon the TMDI equation, we could identify values that were used for comparison with PRSI results in food or food crops potentially consumed by the Brazilian population. To estimate food consumption (F), we used the Dietary Guidelines for the Brazilian people, established by the Brazilian Health Ministry (77), in which food serving portions are recommended. The data on the PR found in the crops were evaluated for each pesticide-active substance.

For comparison purposes, we observed the difference between the TMDI and PRSI from several crops in Brazil in 2019 and 2020. This difference is demonstrated in a percentage higher than the limit tolerated (% higher than the tolerated column, Tables 2, 3). We found that in 2019, all 46 crops analyzed had higher active substance residues than advocated by law, and 38 of these crops displayed 100% more residues than tolerated. The numbers are even worse for 2020 crops, as from 104 crops evaluated, 102 showed 100% more residues than allowed. These findings should be awareness-raising, as several food crops in Brazil were found with increased pesticide residues, often overpassing 1,000%.

It emphasizes the adverse effects that public policies demeaning can have, as Brazil is going against the world and has been increasing the number of legally pesticide-active substances in the country. The draft bill PL 6299/2022 (78)—more widely known as the “poison package,” is an example, already approved by the parliament and waiting to be voted on by the senate (According to the project PL 6299/2002). The demeaning of these public policies not just impacts the overuse of these compounds but also the indiscriminate application of them, as we observe a lot of active substances applied in Brazilian crops are banned for crop use, according to the country's legislation (Supplementary Tables S1, S2).

One example of the importance of sound policies related to pesticides is the latest European Food Safety Authority report, where more than 88.000 food samples produced in 2020 were analyzed, and 94.9% of the samples were within legally permitted levels (79).

Therefore, the dietary risk assessment analysis suggests that the food commodities analyzed are unlikely to concern consumers' health.

The most widely used chemical herbicide is N-(phosphonomethyl) glycine, commonly known as glyphosate (80). In our database, we observed glyphosate as the most used pesticide, applied in eight different regions in 2019 and 43 crops from other areas in 2020, followed by glufosinate. Glyphosate was also the main pesticide used irregularly, i.e., above the maximum allowed residue levels. Economically, this herbicide is popularly sold under the name of Roundup. As a broad-spectrum herbicide, it is used in agriculture and forestry, representing one of the most important chemical compounds in use since its release. Although it is less bioavailable than other herbicides, glyphosate residue levels may represent a risk to consumers depending on several factors, such as the application technique, water quality, and environmental conditions (81).

Glyphosate is considered “safe” because neither its active substance nor its primary degradation product, aminomethylphosphonic acid (AMPA), is associated with any known adverse effect on human health. However, there is a controversy in the literature regarding its carcinogenic potential, as some studies describe its potential to cause endocrine and/or microbiome disruption (80, 82, 83). Besides, glyphosate exposure can also induce epigenetic modulation, such as decreasing global DNA methylation and promoting histone modification, as reviewed elsewhere (84).

Carbendazim (methyl 2-benzimidazolecarbamate) was also detected above the maximum allowed residual level in different crops in 2019 (pineapple, lettuce, papaya, and pear) and 2020 (pineapple). The Brazilian National Health Surveillance Agency (Anvisa) decided to ban the use of carbendazim in 2022, as it was considered carcinogenic (85). Carbendazim is a systemic fungicide that inhibits microtubule polymerization in cells by acting with β-tubulin (86). This inhibition disrupts the microtubule assembly and leads to impaired segregation of chromosomes during cell division, inducing mitotic arrest (87). Organophosphorous pesticide exposure can cause severe systemic and central nervous system disturbances, primarily associated with inhibiting acetylcholinesterase activity (88). Acephate (O, S-dimethyl-acetyl-phosphoramidothioate) and methamidophos (O,S-dimethyl phosphoramidothioate) are two of the most common and efficient OPs used in agriculture. Acephate is classified as a class II “moderately hazardous” pesticide, and methamidophos is classified as a class Ib “highly hazardous” pesticide (89). Acephate is prohibited in tomato crops but was detected in several samples evaluated. Methamidophos is the toxic metabolite of acephate (90). Despite being banned from Brazil since 2012, we observed contamination above the limits of methamidophos in potato crops in 2020. Both active substances have their use restricted or prohibited in the European Union due to their harmful potential, however, we still found residues of these substances, even under restriction or prohibition by law, in food, water, and crops in Brazil.

### Cancer risk evidences for PARA and PNCRC reported pesticides residues

#### Literature screening

Concerning the literature review, we conducted a literature search using the R software (91) with the bibliometrix package (92) to check the terms “pesticides” and “cancer” and “tumor” and “carcinogenesis” in the PubMed database. The search retrieved 174 articles that met our criteria. These terms are appearing more and more in high-impact journals that are devoted to toxicology or cancer studies (Supplementary Figure S1A). Furthermore, an increase in the number of publications dealing with “cancer and pesticides” is evident in recent years (Supplementary Figure S1B).

Brazil is in the top 10 when we look at the countries with higher article production in the last 10 years; the publication rate is increasing in all countries. The USA and China are the countries with the most significant number of publications. Brazil, in this ranking, occupies ninth place with 43 articles available in the PubMed database (Supplementary Figure S2A). Also, a large number of collaborations between different countries to study the topic in question is striking, thus evidencing the concern with the relation between pesticides and cancer (Supplementary Figure S2B).

The trend topics are diverse, but the terms “exposure,” “cancer,” “human,” “pesticides,” and “carcinogenesis” are highlighted. Besides, there is a direct link among each other, meaning the co-occurrence of those terms. These terms were searched for in the titles of the articles (Supplementary Figure S3).

Selected data are discussed in the following topics.

#### Human exposure data

Most of the pesticides reported in PARA and PNCRC are classified by IARC as possibly, potentially or proven carcinogenic (Supplementary Table S3), the most used pesticides as active ingredients and basic formula, chemical group, class, agricultural use, classification according to EPA, classification according to IARC, and classification according to WHO (89).

The mechanism of action of pesticides on target-specific pests is well-established in the literature (93–95), but the action of these compounds on human health is a reasonable investigation and needs to be elucidated. The association with human diseases, including cancer, when exposed to pesticides is already well-established. Still, the mechanisms by which these compounds are responsible for human carcinogenesis must be better understood.

The carcinogenic process can occur gradually, taking several years for a single cancer cell to develop and give rise to a tumor. For tumor development, the cell goes through several phases of growth and adaptation, which can be synthesized in three stages: initiation, promotion, and progression (96).

Initiation is the first phase of tumor development. At this stage, the initiator molecules (carcinogenic) meet the cellular microenvironment and lead to DNA damage, which is not adequately repaired, thus establishing mutations. The greater the exposure to these initiator molecules, the greater the risk of tumor development (97). Promotion is the second phase, affecting cells that have already started (mutants). Promoting agents have the role of increasing the proliferative rate, creating a more significant number of mutation-bearing cells. Promoting agents do not directly affect DNA but cell receptors, leading to the alteration of signaling pathways and increased cell proliferation. Promoters can be further divided into two categories: specific promoters, which interact with receptors on target cells, and non-specific promoters, which alter gene expression without the involvement of a known receptor. Promoters do not lead to the formation of tumors alone; they only increase the cellular expansion of cells already initiated, thus leading to the formation of tumors (98).

The third and final phase of carcinogenesis is cell progression. This phase is associated with changes in the cell genotype, an increase in the rate of proliferation, invasive and metastatic capacity, biochemical (glycolytic pathway and oxidative phosphorylation), and morphological changes (99). At this level of development, tumor formation is irreversible.

IARC has been assessing the carcinogenic risk of pesticides to humans and has critically evaluated monographs on individual chemicals, classifying them into risk cancer categories (IARC53). Carcinogenic risk means the probability that an agent will lead to cancer (neoplasm or tumor) in humans exposed to it. Carcinogen denotes an agent or mixture capable of increasing the incidence of malignant neoplasms. Assessment of carcinogenicity is based on evidence from epidemiological studies, depending on variability over time and location of mixtures, processes, occupations, and industries.

Human exposure to these compounds occurs acutely or chronically and can occur through the skin, respiratory and oral routes, food, water, or accidental ingestion (Table 4) (2, 6, 152). In addition, people may be in direct contact with pesticides during the preparation and use of pesticides and/or indirectly through breathing residual concentrations in the air or exposure to residues found on surfaces, food, and dust (100). Children are vulnerable to pesticides because of their physiological and behavioral differences compared to adults, such as hand-to-mouth exposure (101).

Considering the pesticides described in the PARA and PNRCN reports, we bring some information about their cancer-related effects. Parental occupational exposure to pesticides, such as permethrin, acephate, phosmet, and propiconazole, cause changes in their germ cells. It has been related to an increased risk of developing cancer in childhood, such as acute lymphoblastic leukemia, retinoblastoma, central nervous system tumors, and cell tumor testicular germ cells in adolescence (29, 30, 43, 49, 52). A study carried out by Lombardi et al. (40) related dimethoate and propiconazole to an increased risk of developing medulloblastoma in children whose mothers were exposed to the action of these pesticides during pregnancy (40).

The risk intensifies when the mother is exposed during pregnancy and in the first years of the child's life through contamination by air, dust, and clothes used by the parents when applying pesticides, food, and even breast milk. Exposure does not need to be high or extended because their physiological characteristics make them more susceptible to the effects on their body (102). Among these characteristics, the following can be mentioned: absorption through the skin, which is more intense due to the weight/body surface ratio; greater inhalation due to its respiratory rate and ventilation per minute; higher intake of contaminated food and water per body weight compared to adults and incomplete metabolism causing toxicity to the organism (103).

In human studies, aminomethylphosphonic acid (AMPA), chlorpyrifos, and imazalil were positively associated with the risk of breast cancer (18, 32, 37). It is known that some pesticides accumulate in adipose tissue and can act in the body as endocrine disruptors, having estrogenic effects, which is one of the critical factors that contribute to the development of breast cancer. They can influence the synthesis, transport, metabolism, and elimination of estrogen, disrupting the body's normal homeostasis (37).

Organochlorines act as alpha estrogen nuclear receptor agonists, promoting cell proliferation and tumor progression. Other pesticides promote the activation of cytochrome P450 (CYP) member, CYP19 alpha-aromatase enzyme in adipose tissues, indirectly contributing to the increase of estrogen in peripheral tissues and the intratumoral environment (104). Another mechanism would be related to the interaction with aryl hydrocarbon receptors (AhR). This transcription factor regulates enzymes that participate in the metabolism of xenobiotics belonging to the CYP family. This alteration would lead to the accumulation of adducts in the DNA, one of the factors linked to breast carcinogenesis (105).

A positive association was found between chlorpyrifos exposure and lung cancer incidence (38). Other pesticides, such as acetamiprid, clothianidin, and thiamethoxam, were associated with a higher risk of liver cancer (31). In a case-control study carried out with rural workers exposed to pesticides, the tumor biomarkers p53, alpha-fetoprotein, and alpha L-fucosidase were at higher levels when compared to the unexposed control group. Further, the shorter length of the telomeres and decreased telomerase activity were associated with increased DNA damage (106).

There was a link between the increased risk of developing non-Hodgkin's lymphoma and human exposure to glyphosate (44, 45). Several mechanisms triggered by this exposure may contribute to the onset of the disease, such as immunotoxicity, genotoxicity, and hormonal effects (107). Chromosomal aberrations, such as translocations, would be one of the critical effects caused by pesticides in this type of tumor, favoring the overexpression of oncogenes and thus promoting cell proliferation (108).

The use of permethrin has been linked to the occurrence of multiple myeloma (50, 51, 55). Permethrin can act directly on the progression of the disease by having an immunomodulatory effect or, in the same way, lead to monoclonal gammopathy of undetermined significance, which increases the risk of developing multiple myeloma (109). In the study by Shearer et al. (55), a change in myeloid lineage cells was observed, including immature granulocytes and red blood cells. Consequently, the exacerbated presence of immature granulocytes suppressed the antitumor immune response and favored tumor angiogenesis (55).

Individuals exposed to glyphosate, phosmet, and permethrin are more likely to develop leukemia, and the exposure of pregnant women also increases the chances of their children presenting the disease (43, 46, 52). Some pesticides, such as permethrin, can lead to chromosomal rearrangements, which may later inactivate the topoisomerase 2 or cause oxidative stress, promoting breaks in DNA double-strand (49, 110). The presence of polymorphisms in enzymes such as the glutathione S-transferase and CYP450 families alter their normal functioning, compromising the metabolism of xenobiotics, which may also contribute to increased susceptibility to leukemia (111). Chlorpyrifos and carbofuran were associated with an increased risk of prostate cancer in exposed men (34, 36).

Colorectal and renal tumors were associated with a greater chance of developing in workers exposed to the pesticide chlorpyrifos (35, 39). In turn, astrocytoma had a greater chance of occurrence in those exposed to methyl-Kresoxim (40).

Occupational exposure to omethoate demonstrated changes in the length of telomeres (48). The telomeric region of the chromosome is responsible for preventing the degradation of the final portion of chromosomes and end-to-end chromosomal fusion, ensuring genome stability during cell divisions. Changes in this region contribute to several diseases, including cancer, due to oxidative stress and immunotoxicity that generate DNA damage (112).

#### *In vitro* and *in vivo* data

Pesticide-induced effects have been the subject of many published *in vitro* and *in vivo* studies aimed at expanding the scientific basis of current risk assessment procedures by allowing a better understanding of the mechanism of chemical-induced toxicity and its safety levels. These experimental studies show that pesticides alter DNA, leading to mutations and chromosomal aberrations that lead to the development of cancers and other diseases (2). The articles in our search that address *in vitro* and *in vivo* studies used for this review are listed in Tables 5, 6.

**Table 6:** Mechanisms associated with carcinogenesis *in vivo* after exposure to pesticides.

**Pesticide**	**Type of cancer**	**Mechanism**	**Exposition**	**References**
Cypermethrin	Liver cancer	Cypermethrin treatment suppressed LPS-induced M1 macrophage polarization and promoted a switch to M2 macrophage status. Furthermore, cypermethrin induced metastasis of lung cancer cells in both studies.	–	(113)
Cyproconazole	Liver cancer	Treatment with propiconazole induced liver cell proliferation in an *in vivo* model. Furthermore, TGF-β was overexpressed after treatment.	–	(114)
Cyproconazole	Liver cancer	Cyproconazole induced mild and duration-dependent hepatic hypertrophy in constitutive androstane receptor knockout (CARKO) mice.	–	(115)
Glyfosate	Multiple myeloma	Glyphosate induces monoclonal gammopathy of undetermined significance and promotes disease progression to MM.	Orally	(116)
Glyfosate	Liver cancer	Glyphosate promoted genetic modulation in female Sprague-Dawley rats. There was alteration in the expression of hepatic genes, DNA damage and activation of the TP53 gene.	Orally	(69)
Imazalil	Liver cancer	Imazalil activates the PXR receptor and induces hepatocyte proliferation.	Orally *in vitro*	(117)
Metidathione	Liver	Metidathion increases the incidence of liver toxicity, in addition to increasing neoplasms in male mice.	7 and 28 day exhibitions	(118)
Permethrin	Liver cancer	Permethrin induces a significant increase in hepatocellular neoplasms.	–	(119)
Pyraclostrobin	–	Pyraclostrobin induces elevated levels of hydrogen peroxide, 2, malondialdehyde (MDA) and reactive oxygen species (ROS).	–	(120)
Pyraclostrobin	–	Interaction with pro-apoptotic (Bax), apoptotic (Caspase-3, Caspase-8 and Caspase-9), pro-inflammatory (NFκB), cancer (CYP2E1) and cell regulatory (p53) genes and decreased anti-inflammatory gene expression apoptotic (Bcl-2).	–	(121)
Propiconazole	Liver cancer	Inhibition of CYP450 enzymes, genetic alterations caused by the increase of ROS and promotion of cell proliferation, alterations in DNA directly or indirectly by the action of ROS, promotes proliferation and loss of function of tumor suppressor genes.	–	(122)
Propiconazole	Liver cancer	Propiconazole can induce tumors by a mechanism dependent on constitutive androstane receptors (CAR).	–	(123)
Propiconazole	Liver cancer	Propiconazole affects CYP450, Glutathione S transferase and increases oxidative stress.	Diet	(114)
Propiconazole	Liver cancer	Propiconazole induces an increase in ROS and alters the expression of antioxidant enzymes (SOD, CAT, GST).	Environmental	(124)
Propiconazole	Liver cancer	Propiconazole activates CAR/RXR, P450 metabolism, hepatic hypertrophy-glutathione depletion, LPS/IL-1-mediated inhibition of RXR, and NRF2-mediated oxidative stress pathways.	–	(125)
Propiconazole	Liver cancer	Increased endogenous DNA adducts (carcinogenic DNA-binding molecule) and increased cell proliferation.	Diet	(126)
Propiconazole	Liver cancer	This pesticide activates the CAR receptor and leads to increased liver weight and hepatocyte proliferation.	–	(127)
Thiamethoxam		A study carried out on Drosophila evaluated the pro-mutagenic potential of this pesticide at high concentrations	–	(128)

Some pesticides act directly on receptor expression and hormone secretion. The secretion of estrogen is one of the main pathways affected, suggesting a higher risk for women exposed to these pesticides. Imidacloprid and thiamethoxam, pesticides from the neonicotinoid class, increase the expression of the aromatase enzyme cytochrome P450 19 (CYP19), the key to the stimulation of estrogen biosynthesis. This increase is directly related to the increased proliferation of cell lines such as breast cancer lineage Hs578t and adenocarcinoma lineage H295R (72). Cyfluthrin, chlorpyrifos, and glyphosate, the most widely used pesticide globally, act similarly. These increase estradiol (E2) synthesis in adrenocortical carcinoma (H295R) and breast cancer (T47D) cells and increase cell proliferation *via* the estrogen receptors ERα and ERβ. These results indicate that even low concentrations and environmental levels of pesticides cause increased estrogen levels, which, at high levels, are related to potential risk factors for developing, especially, breast cancer in women (58, 59, 61, 66).

There are indications from studies in animal models that pesticides also act on androgen receptors and on specific factors that stimulate the development of liver neoplasms, such as cyproconazole and propiconazole, from the class of conazoles. These pesticides act as essential mediators for increased hypertrophy and tumor initiation, the constitutive androstane receptor (CAR). Along with these pesticides, imazalil, permethrin, and methidathion act on the liver. These increase hepatocyte proliferation; imazalil, by increasing the expression of transforming growth factor alpha (TGF-α) and genes of the cytochrome p450 family, such as Cyp3a11, a target of the pregnane X receptor (PXR), which has its expression increased in liver carcinogenesis or other adverse events in the organ. In contrast, methidathion and permethrin stimulate liver cell proliferation in a PXR and CAR receptor-independent manner (114, 115, 118, 119, 122, 123, 125, 127).

Other mechanisms may also be responsible for changes in cell proliferation, such as changes in the cell cycle and in the expression of factors linked to tumor progression. Omethoate and cypermethrin alter the cell cycle of hypopharyngeal carcinoma (FaDu) and ovarian cancer (BG-1) lineages. These pesticides activate the Akt/GSK-3β/cyclin D1 signaling pathway and regulate the cyclin D1 gene, which is responsible for the transition between G1-S phases of the cycle; thus, the cell cycle is, in turn, stimulated, resulting in increased cell proliferation (60, 70). Cypermethrin also promotes, in mice, macrophage class switches from M1 (pro-inflammatory) to M2 (anti-inflammatory) that act by inhibiting effector T cells. This modulation can promote lung tumor progression (113).

The pesticide triflumuron, *via* hypoxia-induced factor 1α (HIF-1α), induces, in hepatocellular carcinoma (HepG2) cells, migration, invasion, and metastasis. Interestingly, this is the first time HIF-α is responsible for promoting these changes in this type of cancer (76). Another factor influenced by pesticide exposure is vascular endothelial growth factor A (VEGF-A). Increased VEGF-A levels in MCF-7 and MDA-MB-231 breast cancer cells increased specific parameters such as angiogenesis, migration, and cell invasion in these cell lines after chlorpyrifos exposure. These findings reinforce the role of angiogenesis in breast cancer progression, and that pesticide exposure contributes to this process (62, 63).

Oxidative stress is one of the mechanisms involved in the process of carcinogenesis already established, according to the literature, in several types of cancer, including childhood leukemias. In this sense, studies have been carried out to understand if there is any influence on pesticide exposure and the generation of oxidative stress. Thus, an investigation conducted with lung cell line A549 observed that the pesticide chlorpyrifos could generate oxidative stress in these cells by activating the NRF2 pathway, a transcription factor. Although NRF2 plays a role in decreasing oxidative stress and inflammation, it has been shown that in some cancers, this factor enables malignant cells to undergo metabolic changes leading to rapid proliferation and, therefore, tumor growth, and this is a possible survival mechanism for tumor cells (64, 129, 130).

Another study involving the pesticide Triflumuron was conducted experimentally in animals and HCT 116 cells. This work aimed to evaluate the genotoxicity of this chemical in the models chosen for the experiment. They observed that triflumuron induced the generation of reactive oxygen species, followed by lipid peroxidation, due to increased levels of malondialdehyde, a pro-oxidative parameter, and activating the antioxidant enzymes, catalase, and superoxide dismutase, in human colon tumor cells (HCT 116). These studies suggest that exposure to these substances, even at low concentrations, can induce oxidative stress, a well-established carcinogenic factor in cancer pathophysiology, including a marker of therapeutic response (75, 129).

Other pesticides have shown pro-carcinogenic effects in animal models. For example, in the zebrafish model, the pesticide pyraclostrobin affected apoptosis-related pathways, cancer, and membrane components, leading to mitochondrial dysfunction and cell apoptosis. It is because it induced the production of reactive oxygen species (ROS) and increased the activity of antioxidant enzymes such as catalase (CAT) and superoxide dismutase (SOD). These findings portend the need for further research into pesticide toxicity in aquatic models (120).

It was also observed once the increase of oxidative parameters, such as MDA, in rats exposed to the insecticide pyraclostrobin and the decrease of antioxidant defenses, DNA damage, and histopathological analysis was also observed in the kidneys and liver of these animals (121).

Among pesticides, glyphosate is a widely used herbicide worldwide. Many researchers aim to understand the relationship of this herbicide with cancer because the product is cytotoxic, even at low concentrations and a short duration of exposure. Stur et al. studying a cell line treated with roundup (composed of glyphosate and surfactants) observed that this compound can induce the production of reactive oxygen species by altering cellular metabolism and mitochondrial oxygen consumption, leading to a sequence of events that culminates in cell death. Oxidative stress is one of the pathways altered by glyphosate, but other pathways suffer interference and are also the target of studies (67, 131).

Exposure to pesticides can also induce the expression of genes involved in carcinogenesis. However, it remains unclear which genes and the mechanism responsible for their triggering, so to elucidate which pathways are stimulated, both *in vitro* and *in vivo* studies are carried out (132).

For example, studies carried out with glyphosate demonstrated through animal and *in vitro* experiments the pathways related to the development of the investigated cancer. In the case of exposure to small doses of glyphosate (0.05%) *in vitro* in the breast cancer cell line MCF-7 and MDA-MB-231, dysregulation of 11 canonical gene pathways was observed. The most essential included cell cycle and DNA damage repair and accumulating mutations, once again demonstrating the role of pesticides in mutagenicity by generating stress and cell cycle dysregulation (67).

While an experiment was carried out in an animal model, in this case with female Sprague-Dawley rats, a change in the expression of liver genes was observed, reflecting the activation of the TP53 gene due to the damage caused to the DNA. Furthermore, there was a decrease in the expression of miR-30 and an increase in the expression of miR-10. Dysregulation in the expression of microRNAs can alter the expression of target genes and disrupt cellular pathways. DNA base methylation is another modification capable of influencing gene expression, and this mechanism was also changed by glyphosate methylation (69). The work carried out by Wang et al. verified that MYC mice treated with glyphosate showed benign monoclonal gammopathy, anemia, and increased plasma cells in the bone marrow and spleen. Such findings place pesticides as a potential risk factor for developing multiple myeloma and non-Hodgkin lymphoma (116).

In research carried out *in vitro* to analyze the consequences of exposure to permethrin em ETV6-RUNX1 and IGH-BCL2 genes in K562 cells (chronic myeloid leukemia cells), induction was found of breakage and fusion of the damaging genes associated with lymphoma development (71). Furthermore, permethrin exposure induced numerical aberrations frequently observed in the metaphase phase (49).

Other results from *in vitro* and *in vivo* exposure to pesticides evaluated in the present review are shown in Tables 5, 6.

## Perspectives and conclusions

Some considerations need to be pointed out about PARA reliability and data validity. Among the positive points of PARA, it should be noted that since its implementation in 2001, the program has been expanded in four dimensions: the number of participating states, number of samples analyzed, types of food analyzed and number of active ingredients researched (21). Although there was no standardization in the presentation of results from the beginning, the reports proved to be more detailed and complete. In the case of the Vegetal PNCRC, there have also been advances, especially from 2019 onwards, when the Ministry of Agriculture, Supply, and Livestock, through inspection actions, began to fine irregularities (133). However, the reports are still strictly technical, issued through ordinances, and not very accessible to the general population.

Considering that Brazil is among the three countries that use pesticides in the world, as well as the significant increase in the number of concessions for registration of pesticides in the country from 2016 onwards (134), official surveillance institutions should pay greater attention to the problem, especially in which refers to the contamination of food by these agrochemicals. The PARA and PNCRC Vegetal methodologies still need to be improved to ensure transparency and transmit greater security to the consumer.

In this sense, in the case of PARA, the number of samples is still low compared to other countries, such as the European Union. Recently, it has involved only 1.38% of Brazilian municipalities, 77 out of 5,568 (13). Another point to be highlighted is that Brazil's two most commercialized active ingredients (glyphosate and 2,4-D), widely used in the production of monocultures, only entered the analysis from 2016 onwards. On the other hand, glyphosate is one of the most detected pesticides in the Vegetal PNCRC, mainly in bean samples (20).

Brazilian researchers have also questioned the fact that the multi-exposure risk assessment is not adopted (135–137) since the reports by PARA and PNCRC Vegetal indicate samples contaminated by more than one active ingredient. Thus, the effects that add up and potentiate should be considered in methodologies for analyzing pesticide residues in food.

It is noteworthy that the publication and dissemination of results could be more problematic in the reports. The focus is on the absence of danger, disregarding that more than half of the total samples have some pesticide residue. Thus, if the sample is considered “satisfactory” for the Brazilian MRLs (which are highly permissive), the impression is that Brazilians are purchasing foods that are perfectly suitable for consumption and are also healthy. It is also not usually publicized that not all active ingredients approved for use in Brazil are monitored. In addition, in 2020, the PARA was suspended due to the COVID-19 pandemic, and no results were released after the 2017–2018 cycle.

It is very important to point out that the exposure to pesticides in Brazil is continuous. It occurs directly for farmers who frequently (138) handle these products and indirectly, through the drift of active ingredients to neighboring areas, as well as the contact of farmers' wives and children with different amounts of pesticides, by having contact with clothing used for work, and even pesticide packaging (139, 140). Children are vulnerable to pesticides because of their physiological and behavioral differences compared to adults, such as hand-to-mouth exposure (101, 141). Urban dwellers are also affected, as the urban water supply and many commercialized foods are already contaminated with pesticides (142).

There is no provision in the Brazilian legislation about the review process of the registration of authorized pesticides, and even today, products banned in other countries are used. Decree No. 4.074/2002 (143) recommends that this review could, in theory, occur at any time, guided by international alerts, new scientific studies, or complaints made by reference institutions under its subsection VI, art. 2. It is also noticed that, even in cases of international alerts, the limited resources available in the agencies or the lawsuits filed by corporations linked to agribusiness, not rarely end up hindering and delaying such reviews, worsening the exposure of the population to pesticides (144).

The MRL is defined as the maximum amount of pesticide residue officially accepted in food as a result of proper application at a specific stage, from its production to consumption, expressed in parts (by weight) of the pesticide or its residues per million parts of food (by weight) (ppm or mg/kg) (145). As for the levels of residues contained in food, they must be below the MRLs, established as references after conducting the necessary toxicological studies. In this context, the issue of maximum residue levels (MRLs) is one of the most relevant for food safety in trade negotiations between countries and companies.

When analyzing the PARA reports, one point that draws attention is that there is a category in which the samples are considered satisfactory when they present pesticide residues within a maximum residue limit pre-established through federal government regulations and the Codex Alimentarius. In general, 30–40% of the samples analyzed in each report fall into this classification. However, setting these limits ends up disregarding essential factors such as the joint action of several chemical compounds acting simultaneously in the human body (146), differences in susceptibility according to age and genetic factors, and the effects of chronic exposure (33).

Pesticide exposures in Brazil violate many human rights of the population. The right to life is potentially violated when pesticides contaminate food and water for human consumption. Bodies become ill (147), and the biodiversity of ecosystems is also threatened.

The Brazilian Constitution provides in its article 225 (148) that everyone has the right to an ecologically balanced environment, an asset for shared use by the people and essential to a healthy quality of life, imposing on the government and the community the duty to defend and preserve it for present and future generations. However, Brazil has adopted a position contrary to several countries that start from the precautionary principle concerning pesticides, such as those belonging to the European Union.

Approximately 80% of the pesticides authorized in Brazil are not permitted for use in at least three countries of the Organization for Economic Cooperation and Development (OECD), including countries with agriculture as an essential economic activity. Australia has 40% of its agricultural territory, a similar condition to Brazil, and no records of 114 active ingredients of pesticides allowed in the Brazilian territory were found. Although Brazil and India have relatively close soil and climate conditions, more than 50% of the pesticides that are registered in the first country are not allowed in the second, and the list of active ingredients of pesticides authorized in Brazil includes examples with recognized toxicity on human health and the environment. It extends to the 279 active chemical ingredients for agricultural use registered in Brazil with their regulatory status in the European Union, the United States, Canada, and Japan, which exposes massive differences. While in the European Union, 136 substances registered in Brazil are approved (143 are not approved), in the United States and Canada, 218 substances are approved. In Japan, 205 active ingredients registered in Brazil are approved (149).

Among the most used pesticides in Brazil, glyphosate stands out. In Brazil's regulations, glyphosate has a maximum residue limit of 1 mg/kg in coffee and sugar cane and 10 mg/kg in soy, corresponding to 10, 20, and 200 times the values allowed in the European Union for the same foods. In the human body, glyphosate is detected in blood, breast milk, and urine, with urinary levels in the general population of 0.16–7.6 μg/L, while in the occupationally exposed population, it is 0.26–73.5 μg/L (150, 151).

The European legislation establishes rules for the use and limits of pesticide residues and practices to be incorporated in the member countries of the European Union to gradually reduce the use of pesticides, as well as the use of alternatives that replace the use of chemicals, aiming to protect human and animal health and the environment. These limits are also extended to countries that intend to export to the European Union.

Currently, Bill 6.299/2002 (78) is being processed in the National Congress, already approved in the House of Representatives, which aims to further relax the legislation on pesticides (152) by facilitating the registration of active ingredients known to be prohibited in other countries, among other serious proposals that favor the indiscriminate use. Among the proposed changes is removing the registration prohibition criteria for potentially carcinogenic agents, toxic to the reproductive system, endocrine disruptors, and teratogenic agents, which are currently similar to the requirements adopted in Europe. With the changes, the use of substances associated with these effects may be permitted, subject to risk assessment. In Europe, there is also pressure on this provision. Still, studies have shown that the supposed economic losses would not be more significant than the health costs, loss of individual quality of life, deaths, and reduced productivity due to absenteeism, among others (153). In addition to its various effects, endocrine disruption indicates prohibition in the European Community. However, this device meets resistance to being fully implemented due to the controversies and doubts produced by the economic sectors to define the criteria for this classification, common strategies regarding the regulation, and use of toxic substances (154).

It is important to note that this bill is being processed even after opposing manifestations of Brazilian official technical bodies (155) such as the Brazilian Institute of Environment and Renewable Natural Resources, the National Health Surveillance Agency, the National Cancer Institute, and the Ministry of Labor.

The observations and recommendations of entities linked mainly to health and the environment were ignored by 2/3 (two-thirds) of the parliamentarians of the House of Representatives, who voted in favor of the continuity of the bill's passage (according to project PL 4166/12). It shows that economic interests, high productivity, and profitability are prioritized to the detriment of the population's quality of life.

Regarding food contamination, there is no direct association in the literature between exposure to pesticides and cancer development. However, pesticides such as glyphosate can cause disruptions in several biological pathways that may be linked to carcinogenesis. Glyphosate was our evaluation's most widely applied active ingredient on crops during 2019 and 2020.

Contact with glyphosate can occur *via* the oral, respiratory (pulmonary), or dermal route (156). The dermal route is the complaint of workers exposed to glyphosate by the absorption route of this element (157). Its accumulation in the body is found mainly in the liver, kidneys, colon, and small intestine, and its excretion happens through about 90% in the feces and within 48h in the urine (156). Importantly, even with many studies already confirmed and still being investigated, the ubiquitous cause of glyphosate and its health safety is of great concern (158).

In 2015, the International Agency for Research on Cancer (IARC), part of the World Health Organization, published its carcinogenicity assessment of glyphosate, concluding that this pesticide would likely be carcinogenic to humans (group 2A) based on limited epidemiological evidence in humans, primarily for non-Hodgkin's lymphoma, and significant evidence of carcinogenicity in animals (159, 160), operating through two critical pathways of known human carcinogens, specifically genotoxicity and oxidative stress induction.

Much research verifies glyphosate use and cancer incidence (46). The IARC evaluation of glyphosate resulted in intense opposition from the pesticide industry and led to many industry-sponsored articles and analyses on this subject (161–169). Notably, two of these studies were conducted in communities that had contact with this herbicide through aerial spraying, and caused DNA damage (170) and micronuclei (171).

Subsequently, the European Food Safety Authority (162) and the US Environmental Protection Agency (172) also reviewed this issue. They found that glyphosate is probably not carcinogenic in humans. Most pesticide regulatory agencies in other countries have followed their lead, suggesting that data sets and methodological differences partially explain these divergent views. However, this topic is complex and beyond the scope of this article.

Thus, it is imperative to have strict policies regarding these chemicals, following each crop's recommendations in class and the number of chemicals used.

## Data availability statement

The original contributions presented in the study are included in the article/Supplementary material, further inquiries can be directed to the corresponding author.

## Author contributions

Conceptualization: JB, TF, MO, PL, GB, MC, BG, JS, TS, SG, LZ, and JM. Methodology: GB, MC, TS, and SG. Validation: LZ and JM. Data curation: MO and PL. Writing—original draft preparation: JB, TF, MO, PL, GB, MC, BG, JS, TS, SG, LZ, JM, FR, and CP. Writing—review and editing: JB, TF, FR, and CP. Supervision: FR and CP. Project administration: CP. All authors have read and agreed to the published version of the manuscript.
