# Supplementary material for: Monitoring residues of pesticides in food in Brazil: A multiscale analysis of the main contaminants, dietary cancer risk estimative and mechanisms associated
Source: Front Public Health. 2023 Feb 22;11:1130893. doi: 10.3389/fpubh.2023.1130893 (PMC9992878; doi:10.3389/fpubh.2023.1130893)
Supplement: Supplementary file 1 [file Data_Sheet_1.pdf]

Supplementary material

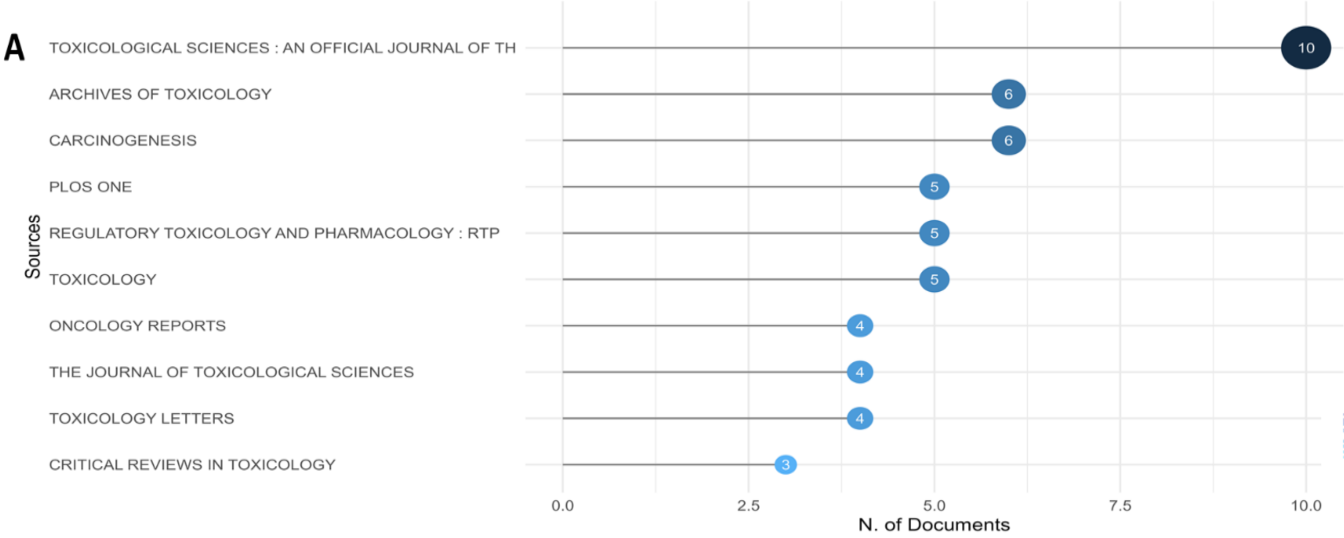

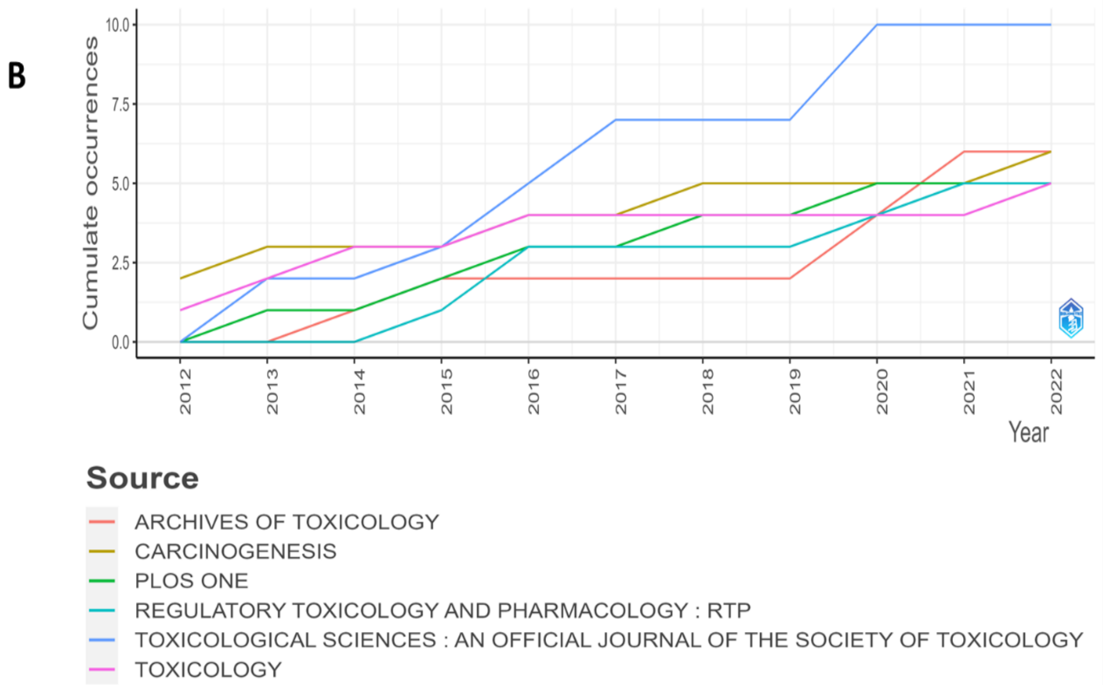

Figure Supplementary.1 (S.1) – Journals in which articles with the topics "pesticides" and "cancer" were published in the last 10 years. **A** – Most Relevant Sources, **B** – Source Growth

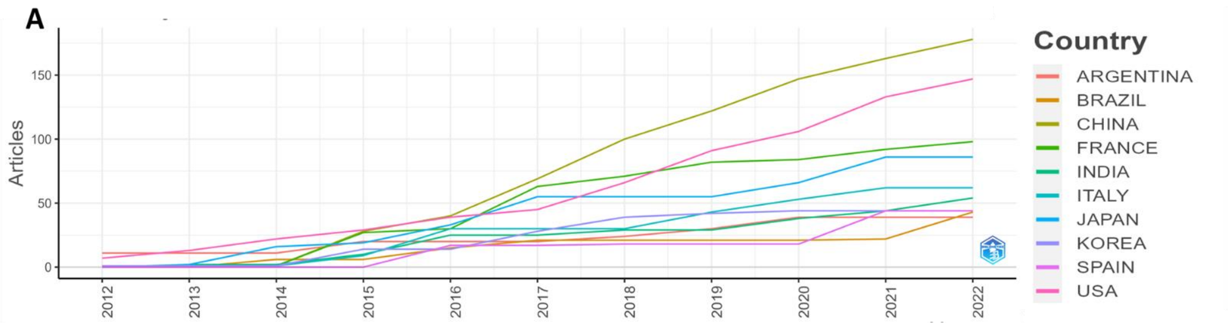

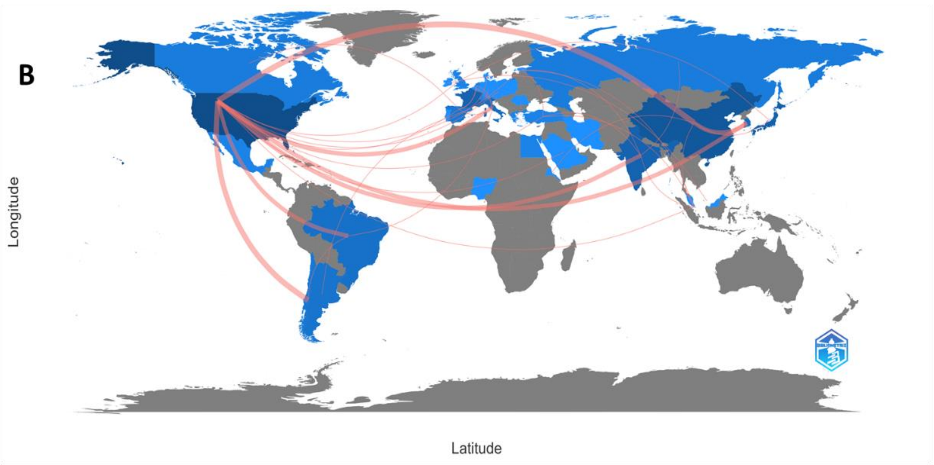

Country	Frequency
CHINA	178
USA	147
FRANCE	98
JAPAN	86
ITALY	62
INDIA	54
SOUTH KOREA	44
SPAIN	44
BRAZIL	43
ARGENTINA	39

Figure Supplementary 2 (S.2) - Article production rate and worldwide collaboration network. **A** - Country Production over Time. **B** - Country collaboration map.

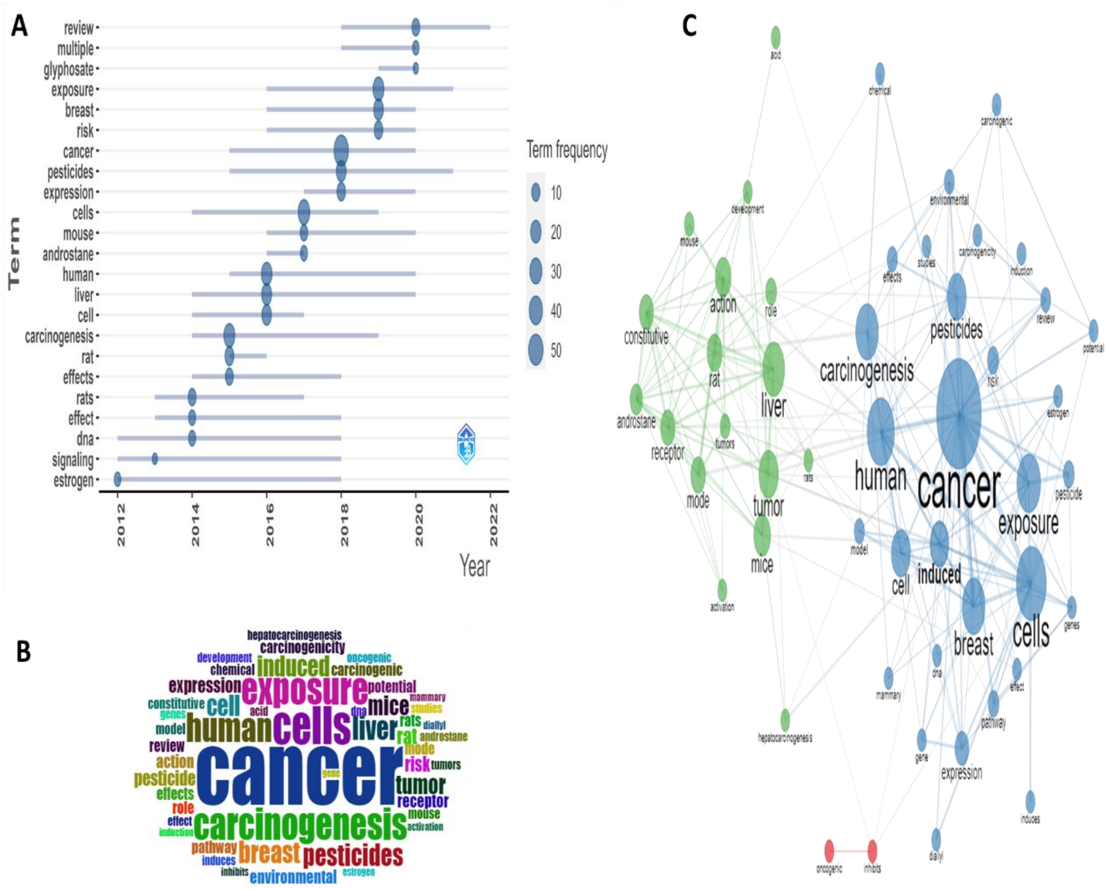

Figure Supplementary (S.3) – Most frequent words found in article's title. **A** – Trend Topics; **B –** Word-Cloud; **C** – Co-occurrence network.

Table Supplementary 4 (S.4): TMDI calculations and PRSI based on analyzes carried in food samples in 2019 with banned active substances.

**Year 2019**							
**Crop**	**Pesticide**	**MLR**	**PR**	**F**	**TMDI**	**PRSI**	**TMDI X PRSI**
Pinneaple	Cyfluthrin	Banned for crop use	0,05	0,13	Banned for crop use	Banned for crop use	Banned for crop use
	Cyfluthrin	Banned for crop use	0,04	0,13	Banned for crop use	Banned for crop use	Banned for crop use
	Cyfluthrin	Banned for crop use	0,04	0,13	Banned for crop use	Banned for crop use	Banned for crop use
	Trifloxystrobin	0,3	0,59	0,13	0,039	0,0767	0,0377
	Carbendazim	0,5	2,34	0,13	0,065	0,3042	0,2392
	Permethrin	Banned for crop use	0,05	0,13	Banned for crop use	Banned for crop use	Banned for crop use
	Carbendazim	0,5	1,89	0,13	0,065	0,2457	0,1807
	Carbendazim	0,5	1,44	0,13	0,065	0,1872	0,1222

	Azoxystrobin	Banned for crop use	0,62	0, 13	Banned for crop use	Banned for crop use	Banned for crop use
	Dimethoate	Banned for crop use	0,01	0, 13	Banned for crop use	Banned for crop use	Banned for crop use
	Dimethoate	Banned for crop use	0,02	0, 13	Banned for crop use	Banned for crop use	Banned for crop use
	Chlorantraniliprole	Banned for crop use	0,01	0, 13	Banned for crop use	Banned for crop use	Banned for crop use
Lettuce	Carbendazim	Banned for crop use	0,04	0, 12	Banned for crop use	Banned for crop use	Banned for crop use
	Cyazofamid	0,2	2,41	0, 12	0,024	0,2892	0,2652
	Kresoxim-methyl	Banned for crop use	1,35	0, 12	Banned for crop use	Banned for crop use	Banned for crop use
	Metalaxyl-M	0,5	0,99	0, 12	0,06	0,1188	0,0588
	Pyraclostrobin	1	3,5	0, 12	0,12	0,42	0,3
	Carbendazim	Banned for crop use	0,02	0, 12	Banned for crop use	Banned for crop use	Banned for crop use
	Cyazofamid	0,2	0,87	0, 12	0,024	0,1044	0,0804
	Kresoxim-methyl	Banned for crop use	0,36	0, 12	Banned for crop use	Banned for crop use	Banned for crop use
Cocoa almond	Cadmium	500	766,15	N A	NA	NA	NA
Banana	Bifenthrin	0,02	0,06	0, 08 6	0,00172	0,00516	0,00344
Beetroot	Acephate	Banned for crop use	0,06	0, 04 3	Banned for crop use	Banned for crop use	Banned for crop use
	Chlorfenapyr	Banned for crop use	0,01	0, 04 3	Banned for crop use	Banned for crop use	Banned for crop use
Carrot	Acephate	Banned for crop use	0,01	0, 04 3	Banned for crop use	Banned for crop use	Banned for crop use
	Chlorfenapyr	Banned for crop use	0,04	0, 04 3	Banned for crop use	Banned for crop use	Banned for crop use
	Cadusafos	Banned for crop use	0,01	0, 04 3	Banned for crop use	Banned for crop use	Banned for crop use
	Cadusafos	Banned for crop use	0,03	0, 04 3	Banned for crop use	Banned for crop use	Banned for crop use
	Chlorpyrifos	Banned for crop use	0,01	0, 04 3	Banned for crop use	Banned for crop use	Banned for crop use
Black bean	Glyphosate	0,05	0,24	0, 08	0,004	0,0192	0,0152
	Glufosinate	0,05	0,41	0, 08	0,004	0,0328	0,0288
	Glyphosate	0,05	0,25	0, 08	0,004	0,02	0,016
	Glyphosate	0,05	0,32	0, 08	0,004	0,0256	0,0216
	Glyphosate	0,05	1,9	0, 08	0,004	0,152	0,148

	Glyphosate	0,05	0,15	0,08	0,004	0,012	0,008
	Glyphosate	0,05	0,14	0,08	0,004	0,0112	0,0072
	Glyphosate	0,05	1,59	0,08	0,004	0,1272	0,1232
	Glufosinate	0,05	0,21	0,08	0,004	0,0168	0,0128
	Glufosinate	0,05	0,3	0,08	0,004	0,024	0,02
	Glufosinate	0,05	0,4	0,08	0,004	0,032	0,028
	Glufosinate	0,05	0,32	0,08	0,004	0,0256	0,0216
	Glufosinate	0,05	0,65	0,08	0,004	0,052	0,048
	Glyphosate	0,05	0,31	0,08	0,004	0,0248	0,0208
Cowpea bean	Glyphosate	Banned for crop use	1,83	0,048	Banned for crop use	Banned for crop use	Banned for crop use
	Glyphosate	Banned for crop use	0,88	0,048	Banned for crop use	Banned for crop use	Banned for crop use
	Glyphosate	Banned for crop use	8,72	0,048	Banned for crop use	Banned for crop use	Banned for crop use
	Glyphosate	Banned for crop use	2,07	0,048	Banned for crop use	Banned for crop use	Banned for crop use
	Glufosinate	0,05	0,1	0,048	0,0024	0,0048	0,0024
	Glyphosate	Banned for crop use	12,51	0,048	Banned for crop use	Banned for crop use	Banned for crop use
	Glyphosate	Banned for crop use	0,56	0,048	Banned for crop use	Banned for crop use	Banned for crop use
	Glyphosate	Banned for crop use	0,92	0,048	Banned for crop use	Banned for crop use	Banned for crop use
	Glyphosate	Banned for crop use	1	0,048	Banned for crop use	Banned for crop use	Banned for crop use
	Glyphosate	Banned for crop use	0,43	0,048	Banned for crop use	Banned for crop use	Banned for crop use
	Glyphosate	Banned for crop use	1,1	0,048	Banned for crop use	Banned for crop use	Banned for crop use
	Glyphosate	Banned for crop use	1,45	0,048	Banned for crop use	Banned for crop use	Banned for crop use
	Glyphosate	Banned for crop use	4,2	0,048	Banned for crop use	Banned for crop use	Banned for crop use
	Glyphosate	Banned for crop use	3,27	0,048	Banned for crop use	Banned for crop use	Banned for crop use
	Glyphosate	Banned for crop use	7,46	0,048	Banned for crop use	Banned for crop use	Banned for crop use

	Glyphosate	Banned for crop use	8,07	0,048	Banned for crop use	Banned for crop use	Banned for crop use
	Glyphosate	Banned for crop use	9,24	0,048	Banned for crop use	Banned for crop use	Banned for crop use
	Glyphosate	Banned for crop use	13,2	0,048	Banned for crop use	Banned for crop use	Banned for crop use
Guava	Cypermethrin	Banned for crop use	0,19	0,095	Banned for crop use	Banned for crop use	Banned for crop use
	FOSMETE	Banned for crop use	0,07	0,095	Banned for crop use	Banned for crop use	Banned for crop use
	Dimethoate	Banned for crop use	0,04	0,095	Banned for crop use	Banned for crop use	Banned for crop use
	Thiamethoxam	Banned for crop use	0,04	0,095	Banned for crop use	Banned for crop use	Banned for crop use
	Carbendazim	Banned for crop use	0,01	0,095	Banned for crop use	Banned for crop use	Banned for crop use
	Cypermethrin	Banned for crop use	0,11	0,095	Banned for crop use	Banned for crop use	Banned for crop use
	Thiamethoxam	Banned for crop use	0,01	0,095	Banned for crop use	Banned for crop use	Banned for crop use
Kiwi	Imazalil	Banned for crop use	0,02	0,154	Banned for crop use	Banned for crop use	Banned for crop use
	Lufenurom	Banned for crop use	0,02	0,154	Banned for crop use	Banned for crop use	Banned for crop use
	METIDATIONA	Banned for crop use	0,14	0,154	Banned for crop use	Banned for crop use	Banned for crop use
Apple	Triethanolamine	Prohibited	0,08	0,13	Banned for crop use	Banned for crop use	Banned for crop use
Papaya	Carbendazim	0,5	1,24	0,16	0,08	0,1984	0,1184
	FAMOXADONA	Banned for crop use	0,11	0,16	Banned for crop use	Banned for crop use	Banned for crop use
	Trifloxystrobin	0,05	0,13	0,16	0,008	0,0208	0,0128
	Triethanolamine	Prohibited	0,02	0,16	Banned for crop use	Banned for crop use	Banned for crop use
Melon	Cypermethrin	0,02	0,06	0,23	0,0046	0,0138	0,0092
	Chlorpyrifos	Banned for crop use	0,03	0,23	Banned for crop use	Banned for crop use	Banned for crop use
	Propargite	Banned for crop use	0,01	0,23	Banned for crop use	Banned for crop use	Banned for crop use
Strawberry	Acetamiprid	Banned for crop use	0,01	0,24	Banned for crop use	Banned for crop use	Banned for crop use
	Acetamiprid	Banned for crop use	0,43	0,24	Banned for crop use	Banned for crop use	Banned for crop use
	Metalaxyl-M	Banned for crop use	0,01	0,24	Banned for crop use	Banned for crop use	Banned for crop use

	Thiamethoxam	0,1	0,13	0,<br>24	0,024	0,0312	0,0072
	Acetamiprid	Banned for crop use	0,2	0,<br>24	Banned for crop use	Banned for crop use	Banned for crop use
	Acetamiprid	Banned for crop use	0,2	0,<br>24	Banned for crop use	Banned for crop use	Banned for crop use
	Fipronil	Banned for crop use	0,02	0,<br>24	Banned for crop use	Banned for crop use	Banned for crop use
	Imidacloprid	Banned for crop use	0,1	0,<br>24	Banned for crop use	Banned for crop use	Banned for crop use
	Imidacloprid	Banned for crop use	0,03	0,<br>24	Banned for crop use	Banned for crop use	Banned for crop use
	Carbendazim	0,5	1,6	0,<br>24	0,12	0,384	0,264
	Thiamethoxam	0,1	0,16	0,<br>24	0,024	0,0384	0,0144
Pear	Carbendazim	Banned for crop use	0,06	0,<br>13<br>3	Banned for crop use	Banned for crop use	Banned for crop use
	Dimethoate	Banned for crop use	0,08	0,<br>13<br>3	Banned for crop use	Banned for crop use	Banned for crop use
Bell pepper	Acephate	Banned for crop use	0,36	0,<br>06	Banned for crop use	Banned for crop use	Banned for crop use
	Cyproconazole	Banned for crop use	0,01	0,<br>06	Banned for crop use	Banned for crop use	Banned for crop use
	Profenofos	Banned for crop use	0,1	0,<br>06	Banned for crop use	Banned for crop use	Banned for crop use
	Propargite	Banned for crop use	0,01	0,<br>06	Banned for crop use	Banned for crop use	Banned for crop use
	Cypermethrin	0,02	0,04	0,<br>06	0,0012	0,0024	0,0012
	Dimethoate	Banned for crop use	0,03	0,<br>06	Banned for crop use	Banned for crop use	Banned for crop use
	Methomyl	Banned for crop use	0,11	0,<br>06	Banned for crop use	Banned for crop use	Banned for crop use
	Profenofos	Banned for crop use	0,1	0,<br>06	Banned for crop use	Banned for crop use	Banned for crop use
	Propargite	Banned for crop use	0,21	0,<br>06	Banned for crop use	Banned for crop use	Banned for crop use
	Bifenthrin	Banned for crop use	0,12	0,<br>06	Banned for crop use	Banned for crop use	Banned for crop use
	Clothianidin	Banned for crop use	0,06	0,<br>06	Banned for crop use	Banned for crop use	Banned for crop use
	Fenpyroximate	0,1	0,3	0,<br>06	0,006	0,018	0,012
	Metalaxyl-M	Banned for crop use	0,01	0,<br>06	Banned for crop use	Banned for crop use	Banned for crop use
	Flubendiamide	Banned for crop use	0,03	0,<br>06	Banned for crop use	Banned for crop use	Banned for crop use
	Trinexapac-ethyl	Banned for crop use	0,05	0,<br>06	Banned for crop use	Banned for crop use	Banned for crop use
	Hexythiazox	Banned for crop use	0,03	0,<br>06	Banned for crop use	Banned for crop use	Banned for crop use
	Chlorpyrifos	Banned for crop use	0,05	0,<br>06	Banned for crop use	Banned for crop use	Banned for crop use

	Methomyl	Banned for crop use	0,17	0, 06	Banned for crop use	Banned for crop use	Banned for crop use
	Cypermethrin	0,02	0,09	0, 06	0,0012	0,0054	0,0042
	Fenpropathrin	Banned for crop use	0,23	0, 06	Banned for crop use	Banned for crop use	Banned for crop use
	Acephate	Banned for crop use	0,28	0, 06	Banned for crop use	Banned for crop use	Banned for crop use
	Acephate	Banned for crop use	0,03	0, 06	Banned for crop use	Banned for crop use	Banned for crop use
	Chlorpyrifos	Banned for crop use	0,07	0, 06	Banned for crop use	Banned for crop use	Banned for crop use
	Propargite	Banned for crop use	0,03	0, 06	Banned for crop use	Banned for crop use	Banned for crop use
	Cyproconazole	Banned for crop use	0,08	0, 06	Banned for crop use	Banned for crop use	Banned for crop use
	Cypermethrin	0,02	0,14	0, 06	0,0012	0,0084	0,0072
	Dimethoate	Banned for crop use	0,05	0, 06	Banned for crop use	Banned for crop use	Banned for crop use
	Propargite	Banned for crop use	0,27	0, 06	Banned for crop use	Banned for crop use	Banned for crop use
	Acephate	Banned for crop use	1,08	0, 06	Banned for crop use	Banned for crop use	Banned for crop use
	Chlorpyrifos	Banned for crop use	0,01	0, 06	Banned for crop use	Banned for crop use	Banned for crop use
	Triflumurom	Banned for crop use	0,01	0, 06	Banned for crop use	Banned for crop use	Banned for crop use
	Methomyl	Banned for crop use	0,03	0, 06	Banned for crop use	Banned for crop use	Banned for crop use
	Acephate	Banned for crop use	0,59	0, 06	Banned for crop use	Banned for crop use	Banned for crop use
Soy bean	Glyphosate	10	17,1	0, 04 3	0,43	0,7353	0,3053
	Glyphosate	10	30,4	0, 04 3	0,43	1,3072	0,8772
	Cypermethrin	0,05	0,335	0, 04 3	0,00215	0,014405	0,012255
Tomato	Bifenthrin	0,02	0,04	0, 08	0,0016	0,0032	0,0016
	Acephate	0,02	0,03777	0, 08	0,0016	0,0030216	0,0014216
	Acephate	0,02	1,31	0, 08	0,0016	0,1048	0,1032
	Fipronil	Banned for crop use	0,01	0, 08	Banned for crop use	Banned for crop use	Banned for crop use
	Acephate	0,02	0,03	0, 08	0,0016	0,0024	0,0008
	Acephate	0,02	0,09254	0, 08	0,0016	0,0074032	0,0058032
	Acephate	0,02	0,58	0, 08	0,0016	0,0464	0,0448
	Cyromazine	0,03	0,07	0, 08	0,0024	0,0056	0,0032

	Fipronil	Banned for crop use	0,04	0,08	Banned for crop use	Banned for crop use	Banned for crop use
	Acephate	0,02	1,13	0,08	0,0016	0,0904	0,0888
	Fentina	Banned for crop use	0,015	0,08	Banned for crop use	Banned for crop use	Banned for crop use
	Trinexapac-ethyl	Banned for crop use	0,02	0,08	Banned for crop use	Banned for crop use	Banned for crop use
Wheat	Permethrin	0,02	0,34	NA	NA	NA	NA
Grape	Dimethomorph	2	2,75	0,0992	0,1984	0,2728	0,0744

NA: No recommended serving portion information.

Table Supplementary 5 (S.5)- TMDI calculations and PRSI based on analyzes carried in food samples in 2020 with banned active substances.

**Year 2020**							
**Crop**	**Pesticide**	**MLR**	**PRSI**	**F**	**TMDI**	**PRSI**	**TMDI X PRSI**
Pinneapple	Carbendazim	0,5	2,0206	0,13	0,065	0,262678	0,197678
	Carbendazim	0,5	2,68036	0,13	0,065	0,3484468	0,2834468
	Carbendazim	0,5	1,66211	0,13	0,065	0,2160743	0,1510743
	Carbendazim	0,5	1,83862	0,13	0,065	0,2390206	0,1740206
	Carbendazim	0,5	2,44249	0,13	0,065	0,3175237	0,2525237
	Carbendazim	0,5	2,13737	0,13	0,065	0,2778581	0,2128581
	Dimethoate	Banned for crop use	0,09904	0,13	Banned for crop use	0,0128752	Banned for crop use
	Azoxystrobin	Banned for crop use	0,0141	0,13	Banned for crop use	0,001833	Banned for crop use
Banana	Lambda-cyhalothrin	Banned for crop use	0,01418	0,086	Banned for crop use	0,00121948	Banned for crop use
	Triflumurom	Banned for crop use	0,1	0,086	Banned for crop use	0,0086	Banned for crop use
Potatoes	Acephate	0,1	0,24916	0,2025	0,02025	0,0504549	0,0302049
	Imidacloprid	0,05	0,10035	0,2025	0,010125	0,020320875	0,010195875

	Methamidophos	0,01	0,02216	0,2025	0,002025	0,0044874	0,0024624
Carrot	Chlorpyrifos	Banned for crop use	0,01497	0,038	Banned for crop use	0,00056886	Banned for crop use
	Metalaxyl-M	Banned for crop use	0,02848	0,038	Banned for crop use	0,00108224	Banned for crop use
	Profenofos	Banned for crop use	0,01956	0,038	Banned for crop use	0,00074328	Banned for crop use
	Flutriafol	Banned for crop use	0,0142	0,038	Banned for crop use	0,0005396	Banned for crop use
	Acephate	Banned for crop use	0,02413	0,038	Banned for crop use	0,00091694	Banned for crop use
	Acephate	Banned for crop use	0,12701	0,038	Banned for crop use	0,00482638	Banned for crop use
	Chlorpyrifos	Banned for crop use	0,02279	0,038	Banned for crop use	0,00086602	Banned for crop use
	Acephate	Banned for crop use	0,18416	0,038	Banned for crop use	0,00699808	Banned for crop use
Citrus	Carbofuran	Prohibited	0,02	0,137	Prohibited	Prohibited	Banned for crop use
	Profenofos	Banned for crop use	0,11	0,137	Banned for crop use	Banned for crop use	Banned for crop use
Black bean	Glyphosate	0,05	0,55	0,08	0,004	0,044	0,04
	Glyphosate	0,05	0,25	0,08	0,004	0,02	0,016
	Glufosinate	0,05	0,39	0,08	0,004	0,0312	0,0272
	Glyphosate	0,05	0,165	0,08	0,004	0,0132	0,0092
	Glyphosate	0,05	0,76	0,08	0,004	0,0608	0,0568
	Glyphosate	0,05	0,44	0,08	0,004	0,0352	0,0312
	Glufosinate	0,05	0,13	0,08	0,004	0,0104	0,0064
	Glyphosate	0,05	9,35	0,08	0,004	0,748	0,744
	Glyphosate	0,05	0,23	0,08	0,004	0,0184	0,0144
	Glyphosate	0,05	2,3	0,08	0,004	0,184	0,18
	Glyphosate	0,05	0,16	0,08	0,004	0,0128	0,0088
	Glufosinate	0,05	1,02	0,08	0,004	0,0816	0,0776

Glyphosate	0,05	0,73	0,08	0,004	0,0584	0,0544
Glufosinate	0,05	0,3	0,08	0,004	0,024	0,02
Glyphosate	0,05	0,37	0,08	0,004	0,0296	0,0256
Glyphosate	0,05	0,36	0,08	0,004	0,0288	0,0248
Acephate	0,02	0,03	0,08	0,0016	0,0024	0,0008
Glyphosate	0,05	1,01	0,08	0,004	0,0808	0,0768
Glufosinate	0,05	0,1	0,08	0,004	0,008	0,004
Glyphosate	0,05	0,2	0,08	0,004	0,016	0,012
Glyphosate	0,05	0,22	0,08	0,004	0,0176	0,0136
Glyphosate	0,05	3,23	0,08	0,004	0,2584	0,2544
Glyphosate	0,05	1,59	0,08	0,004	0,1272	0,1232
Glyphosate	0,05	0,22	0,08	0,004	0,0176	0,0136
Glufosinate	0,05	0,1	0,08	0,004	0,008	0,004
Glyphosate	0,05	0,13	0,08	0,004	0,0104	0,0064
Glufosinate	0,05	0,14	0,08	0,004	0,0112	0,0072
Glufosinate	0,05	0,39	0,08	0,004	0,0312	0,0272
Glufosinate	0,05	1,01	0,08	0,004	0,0808	0,0768
Glufosinate	0,05	0,18	0,08	0,004	0,0144	0,0104
Glufosinate	0,05	0,12	0,08	0,004	0,0096	0,0056
Glyphosate	0,05	0,3	0,08	0,004	0,024	0,02
Glufosinate	0,05	0,12	0,08	0,004	0,0096	0,0056
Glufosinate	0,05	0,92	0,08	0,004	0,0736	0,0696
Glyphosate	0,05	0,74	0,08	0,004	0,0592	0,0552
Glufosinate	0,05	0,16	0,08	0,004	0,0128	0,0088
Glufosinate	0,05	0,27	0,08	0,004	0,0216	0,0176
Pirimiphos-methyl	Banned for crop use	0,01	0,08	Banned for crop use	Banned for crop use	Banned for crop use
Glyphosate	0,05	4,62	0,08	0,004	0,3696	0,3656

	Glufosinate	0,05	0,97	0,08	0,004	0,0776	0,0736
	Glyphosate	0,05	3,5	0,08	0,004	0,28	0,276
Cowpea bean	Glyphosate	0,01	2,47	0,048	0,00048	0,11856	0,11808
	AMPA (Glyphosate metabolite)	0,01	0,1	0,048	0,00048	0,0048	0,00432
	Glyphosate	0,01	3,09	0,048	0,00048	0,14832	0,14784
	Pirimiphos-methyl	Banned for crop use	0,06	0,048	Banned for crop use	0,00288	Banned for crop use
	Glyphosate	0,01	1,87	0,048	0,00048	0,08976	0,08928
	Acephate	0,02	0,025	0,048	0,00096	0,0012	0,00024
	AMPA (Glyphosate metabolite)	0,01	0,12	0,048	0,00048	0,00576	0,00528
	Glufosinate	0,05	0,27	0,048	0,0024	0,01296	0,01056
	Glyphosate	0,01	0,22	0,048	0,00048	0,01056	0,01008
	Glyphosate	0,01	8,74	0,048	0,00048	0,41952	0,41904
	AMPA (Glyphosate metabolite)	0,01	0,21	0,048	0,00048	0,01008	0,0096
	Glyphosate	0,01	0,8	0,048	0,00048	0,0384	0,03792
	AMPA (Glyphosate metabolite)	0,01	0,08	0,048	0,00048	0,00384	0,00336
	Pirimiphos-methyl	Banned for crop use	0,06	0,048	Banned for crop use	Banned for crop use	Banned for crop use
	Glyphosate	0,01	2,75	0,048	0,00048	0,132	0,13152
	Pirimiphos-methyl	Banned for crop use	0,09	0,048	Banned for crop use	0,00432	Banned for crop use
	AMPA (Glyphosate metabolite)	0,01	0,11	0,048	0,00048	0,00528	0,0048
	Glyphosate	0,01	2,13	0,048	0,00048	0,10224	0,10176
	AMPA (Glyphosate metabolite)	0,01	0,09	0,048	0,00048	0,00432	0,00384
	Glyphosate	0,01	9,1	0,048	0,00048	0,4368	0,43632
	AMPA (Glyphosate metabolite)	0,01	0,35	0,048	0,00048	0,0168	0,01632

	Glyphosate	0,01	0,2	0,048	0,00048	0,0096	0,00912
	Glyphosate	0,01	1,52	0,048	0,00048	0,07296	0,07248
	Glyphosate	0,01	0,28	0,048	0,00048	0,01344	0,01296
	Glufosinate	0,05	0,39	0,048	0,0024	0,01872	0,01632
	Pirimiphos-methyl	Banned for crop use	0,007	0,048	Banned for crop use	Banned for crop use	Banned for crop use
	Glyphosate	0,01	0,749	0,048	0,00048	0,035952	0,035472
	Glyphosate	0,01	2,91	0,048	0,00048	0,13968	0,1392
	AMPA (Glyph osate metabolite)	0,01	0,12	0,048	0,00048	0,00576	0,00528
	Pirimiphos-methyl	Banned for crop use	0,01	0,048	Banned for crop use	0,00048	Banned for crop use
	Glyphosate	0,01	1,5	0,048	0,00048	0,072	0,07152
	Glyphosate	0,01	3,57	0,048	0,00048	0,17136	0,17088
	Glyphosate	0,01	0,65	0,048	0,00048	0,0312	0,03072
	Pirimiphos-methyl	Banned for crop use	0,01	0,048	Banned for crop use	Banned for crop use	Banned for crop use
	Glyphosate	0,05	0,94	0,048	0,0024	0,04512	0,04272
	Glyphosate	0,01	6,82	0,048	0,00048	0,32736	0,32688
	AMPA (Glyph osate metabolite)	0,01	0,21	0,048	0,00048	0,01008	0,0096
	Glyphosate	0,01	0,88	0,048	0,00048	0,04224	0,04176
Bell Pepper	Dimethoate	Banned for crop use	0,01126	0,06	Banned for crop use	Banned for crop use	Banned for crop use
	Propargite	Banned for crop use	0,09607	0,06	Banned for crop use	Banned for crop use	Banned for crop use
	Profenofos	Banned for crop use	0,03295	0,06	Banned for crop use	Banned for crop use	Banned for crop use
	Cypermethrin	0,02	0,05886	0,06	0,0012	0,0035316	0,0023316
	Fipronil	Banned for crop use	0,05473	0,06	Banned for crop use	Banned for crop use	Banned for crop use
	Profenofos	Banned for crop use	0,23322	0,06	Banned for crop use	Banned for crop use	Banned for crop use

Chlorpyrifos	Banned for crop use	0,10076	0,06	Banned for crop use	Banned for crop use	Banned for crop use
Chlorpyrifos	Banned for crop use	0,032	0,06	Banned for crop use	Banned for crop use	Banned for crop use
Flutriafol	0,2	0,41636	0,06	0,012	0,0249816	0,0129816
Propargite	Banned for crop use	0,09861	0,06	Banned for crop use	Banned for crop use	Banned for crop use
Acephate	Banned for crop use	0,0715	0,06	Banned for crop use	Banned for crop use	Banned for crop use
Methomyl	Banned for crop use	0,39236	0,06	Banned for crop use	Banned for crop use	Banned for crop use
Acephate	Banned for crop use	0,23586	0,06	Banned for crop use	Banned for crop use	Banned for crop use
Methomyl	Banned for crop use	0,02953	0,06	Banned for crop use	Banned for crop use	Banned for crop use
Propargite	Banned for crop use	0,01764	0,06	Banned for crop use	Banned for crop use	Banned for crop use
Dimethoate	Banned for crop use	0,05639	0,06	Banned for crop use	Banned for crop use	Banned for crop use
Epoxiconazole	Banned for crop use	0,03539	0,06	Banned for crop use	Banned for crop use	Banned for crop use
Profenofos	Banned for crop use	0,1002	0,06	Banned for crop use	Banned for crop use	Banned for crop use
Chlorpyrifos	Banned for crop use	0,1019	0,06	Banned for crop use	Banned for crop use	Banned for crop use
Propiconazole	Banned for crop use	0,01888	0,06	Banned for crop use	Banned for crop use	Banned for crop use
Acephate	Banned for crop use	1,32415	0,06	Banned for crop use	Banned for crop use	Banned for crop use
Methomyl	Banned for crop use	0,0323	0,06	Banned for crop use	Banned for crop use	Banned for crop use
Profenofos	Banned for crop use	0,24681	0,06	Banned for crop use	Banned for crop use	Banned for crop use
Fenpropatrina	0,2	0,78493	0,06	0,012	0,0470958	0,0350958
Cypermethrin	0,02	0,05847	0,06	0,0012	0,0035082	0,0023082

Profenofos	Banned for crop use	0,27061	0,06	Banned for crop use	Banned for crop use	Banned for crop use
Cypermethrin	0,02	0,07684	0,06	0,0012	0,0046104	0,0034104
Omethoate	Banned for crop use	0,01638	0,06	Banned for crop use	Banned for crop use	Banned for crop use
Methomyl	Banned for crop use	0,01438	0,06	Banned for crop use	Banned for crop use	Banned for crop use
Cypermethrin	0,02	0,09332	0,06	0,0012	0,0055992	0,0043992
Profenofos	Banned for crop use	0,08341	0,06	Banned for crop use	Banned for crop use	Banned for crop use
Metalaxyl-M	Banned for crop use	0,05807	0,06	Banned for crop use	Banned for crop use	Banned for crop use
Methomyl	Banned for crop use	0,05615	0,06	Banned for crop use	Banned for crop use	Banned for crop use
Fipronil	Banned for crop use	0,01421	0,06	Banned for crop use	Banned for crop use	Banned for crop use
Triflumurom	Banned for crop use	0,0203	0,06	Banned for crop use	Banned for crop use	Banned for crop use
Acephate	Banned for crop use	0,64654	0,06	Banned for crop use	Banned for crop use	Banned for crop use
Triflumurom	Banned for crop use	0,05341	0,06	Banned for crop use	Banned for crop use	Banned for crop use
Acephate	Banned for crop use	0,46733	0,06	Banned for crop use	Banned for crop use	Banned for crop use
Cypermethrin	0,02	0,049	0,06	0,0012	0,00294	0,00174
Methomyl	Banned for crop use	0,08175	0,06	Banned for crop use	Banned for crop use	Banned for crop use
Chlorpyrifos	Banned for crop use	0,10568	0,06	Banned for crop use	Banned for crop use	Banned for crop use
Methomyl	Banned for crop use	0,03452	0,06	Banned for crop use	Banned for crop use	Banned for crop use
Acephate	Banned for crop use	0,66217	0,06	Banned for crop use	Banned for crop use	Banned for crop use
Dimethoate	Banned for crop use	0,02158	0,06	Banned for crop use	Banned for crop use	Banned for crop use

Acephate	Banned for crop use	0,35381	0,06	Banned for crop use	Banned for crop use	Banned for crop use
Triflumurom	Banned for crop use	0,02381	0,06	Banned for crop use	Banned for crop use	Banned for crop use
Acephate	Banned for crop use	0,07096	0,06	Banned for crop use	Banned for crop use	Banned for crop use
Acephate	Banned for crop use	0,05817	0,06	Banned for crop use	Banned for crop use	Banned for crop use
Profenofos	Banned for crop use	0,06308	0,06	Banned for crop use	Banned for crop use	Banned for crop use
Bifenthrin	Banned for crop use	0,02467	0,06	Banned for crop use	Banned for crop use	Banned for crop use
Profenofos	Banned for crop use	0,03537	0,06	Banned for crop use	Banned for crop use	Banned for crop use
Acephate	Banned for crop use	0,11123	0,06	Banned for crop use	Banned for crop use	Banned for crop use
Acephate	Banned for crop use	0,018	0,06	Banned for crop use	Banned for crop use	Banned for crop use
Acephate	Banned for crop use	1,70436	0,06	Banned for crop use	Banned for crop use	Banned for crop use
Methomyl	Banned for crop use	0,029	0,06	Banned for crop use	Banned for crop use	Banned for crop use
Acephate	Banned for crop use	0,92042	0,06	Banned for crop use	Banned for crop use	Banned for crop use
Propargite	Banned for crop use	0,52676	0,06	Banned for crop use	Banned for crop use	Banned for crop use
Acephate	Banned for crop use	1,0971	0,06	Banned for crop use	Banned for crop use	Banned for crop use
Propargite	Banned for crop use	0,79297	0,06	Banned for crop use	Banned for crop use	Banned for crop use
Propargite	Banned for crop use	0,14264	0,06	Banned for crop use	Banned for crop use	Banned for crop use
Fenpropatrina	0,2	0,61082	0,06	0,012	0,0366492	0,0246492
Propargite	Banned for crop use	0,08542	0,06	Banned for crop use	Banned for crop use	Banned for crop use
Propargite	Banned for crop use	0,06616	0,06	Banned for crop use	Banned for crop use	Banned for crop use

Fenpropatrina	0,2	0,45142	0,06	0,012	0,0270852	0,0150852
Propargite	Banned for crop use	0,04763	0,06	Banned for crop use	Banned for crop use	Banned for crop use
Propargite	Banned for crop use	0,10867	0,06	Banned for crop use	Banned for crop use	Banned for crop use
Fenpropatrina	0,2	0,49321	0,06	0,012	0,0295926	0,0175926
Propargite	Banned for crop use	0,01466	0,06	Banned for crop use	Banned for crop use	Banned for crop use
Profenofos	Banned for crop use	0,18174	0,06	Banned for crop use	Banned for crop use	Banned for crop use
Acephate	Banned for crop use	0,50216	0,06	Banned for crop use	Banned for crop use	Banned for crop use
Acephate	Banned for crop use	0,66	0,06	Banned for crop use	Banned for crop use	Banned for crop use
Chlorpyrifos	Banned for crop use	0,01	0,06	Banned for crop use	Banned for crop use	Banned for crop use
Acephate	Banned for crop use	0,17	0,06	Banned for crop use	Banned for crop use	Banned for crop use
Chlorfenapyr	0,3	1,55	0,06	0,018	0,093	0,075
Acephate	Banned for crop use	0,02	0,06	Banned for crop use	Banned for crop use	Banned for crop use
Cypermethrin	0,02	0,05	0,06	0,0012	0,003	0,0018
Lufenuron	Banned for crop use	0,11	0,06	Banned for crop use	Banned for crop use	Banned for crop use
Profenofos	Banned for crop use	1,35	0,06	Banned for crop use	Banned for crop use	Banned for crop use
Cypermethrin	0,02	0,75	0,06	0,0012	0,045	0,0438
Triflumurom	Banned for crop use	0,02	0,06	Banned for crop use	Banned for crop use	Banned for crop use
Triflumurom	Banned for crop use	0,04	0,06	Banned for crop use	Banned for crop use	Banned for crop use
Benalaxyl	Banned for crop use	0,02	0,06	Banned for crop use	Banned for crop use	Banned for crop use
Chlorpyrifos	Banned for crop use	0,03	0,06	Banned for crop use	Banned for crop use	Banned for crop use

	Cypermethrin	0,02	0,25	0,06	0,0012	0,015	0,0138
	Triflumurom	Banned for crop use	0,01	0,06	Banned for crop use	Banned for crop use	Banned for crop use
	Propargite	Banned for crop use	0,01031	0,06	Banned for crop use	Banned for crop use	Banned for crop use
Tomato	Acephate	0,02	0,13673	0,08	0,0016	0,0109384	0,0093384
	Bifenthrin	0,02	0,04778	0,08	0,0016	0,0038224	0,0022224
	Lambda-cyhalothrin	0,05	0,12741	0,08	0,004	0,0101928	0,0061928
	Fipronil	Banned for crop use	0,05898	0,08	Banned for crop use	Banned for crop use	Banned for crop use
	Fipronil	Banned for crop use	0,1062	0,08	Banned for crop use	Banned for crop use	Banned for crop use
	Fipronil	Banned for crop use	0,06941	0,08	Banned for crop use	Banned for crop use	Banned for crop use
	Fipronil	Banned for crop use	0,01733	0,08	Banned for crop use	Banned for crop use	Banned for crop use
	Acephate	0,02	0,07091	0,08	0,0016	0,0056728	0,0040728
	Carbosulfan	Banned for crop use	0,01972	0,08	Banned for crop use	Banned for crop use	Banned for crop use
	Acephate	0,02	0,08281	0,08	0,0016	0,0066248	0,0050248
	Lambda-cyhalothrin	0,05	0,10878	0,08	0,004	0,0087024	0,0047024
	Fipronil	Banned for crop use	0,01483	0,08	Banned for crop use	Banned for crop use	Banned for crop use
	Acephate	0,02	0,60658	0,08	0,0016	0,0485264	0,0469264
	FOSMETE	Banned for crop use	0,02101	0,08	Banned for crop use	Banned for crop use	Banned for crop use
	Acephate	0,02	0,24	0,08	0,0016	0,0192	0,0176
Grapes	Propargite	Banned for crop use	0,31871	0,0992	Banned for crop use	Banned for crop use	Banned for crop use
	Hexythiazox	Banned for crop use	0,04037	0,0992	Banned for crop use	Banned for crop use	Banned for crop use
	Propargite	Banned for crop use	0,18689	0,0992	Banned for crop use	Banned for crop use	Banned for crop use

	Hexythiazox	Banned for crop use	0,01788	0,0992	Banned for crop use	Banned for crop use	Banned for crop use
	Dimethoate	Banned for crop use	0,01716	0,0992	Banned for crop use	Banned for crop use	Banned for crop use
	Cyazofamid	0,5	1,1673	0,0992	0,0496	0,11579616	0,06619616
	Propargite	Banned for crop use	0,05006	0,0992	Banned for crop use	Banned for crop use	Banned for crop use
	Propargite	Banned for crop use	0,29473	0,0992	Banned for crop use	Banned for crop use	Banned for crop use

Table Supplementary 6 (S.6): Active ingredients used in Brazil, chemical group, agricultural application, and human carcinogenicity classifications by IARC and WHO.

**Pesticide**	**Formula**	**Chemical group**	**Class**	**Agricultural use**	**IARC Classification**	**WHO Classification**
Acephate	$C_4H_{10}NO_3PS$	O	Insecticide and acaricide	cotton, peanut, potato, citrus, beans, melon, soy, low tomato	not informed	II
Acetamiprid	$C_{10}H_{11}ClN_4$	Neonicotinoid	Insecticide	cotton, beans, tomato	not informed	II
Azoxystrobin	$C_{22}H17N_3O_5$	Strobilurin	Fungicide	cotton, rice, oats, banana, barley, soy, wheat	not informed	U
Benalaxyl	$C_{20}H_{23}NO_3$	Acylalaninate	Fungicide	potato, onion, tomato, and grape	not informed	III
Bifenthrin	$C_{23}H_{22}ClF_3O_2$	Pyrethroid	Insecticide and acaricide	cotton, sugarcane, citrus, chrysanthemum, papaya, mango, melon, rose, soybean, grape	not informed	II
Carbosulfan	$C_{20}H_{32}N_2O_3S$	Benzofuranyl methylcarbamate	Insecticide, acaricide and nematicide	cotton, irrigated rice, potato, papaya, mango, tomato, grape	not informed	II
Cyfluthrin	$C_{22}H_{18}Cl_2FNO_3$	Pyrethroid	Insecticide	cotton, garlic, potato, coffee, onion, citrus, cabbage, beans,	not informed	Ib

				corn, soybean, tomato, wheat		
Zeta-cypermethrin	$C_{22}H_{19}Cl_2NO_3$	Pyrethroid	Insecticide	cotton, rice, irrigated rice, coffee, onion, corn, soybean, tomato	not informed	II
Cyproconazole	$C_{15}H_{18}ClN_3O$	Triazole	Fungicide	cotton, irrigated rice, oats, coffee, sugar cane, barley, eucalyptus, sunflower, corn, soybean, wheat	not informed	II
Cyromazine	$C_6H_{10}N_6$	Triazinamine	Insecticide	potato, chrysanthemum, beans, pod beans, watermelon, melon, cucumber, tomato	not informed	III
Cadusafos	$C_{10}H_{23}O_2S_2P$	Organophosphate	Insecticide and nematicide	cotton, potato, coffee, sugar cane	not informed	Ib
Chlorfenapyr	$C_{15}H_{11}BrClF_3N_2O$	Pyrazole analogue	Insecticide and acaricide	cotton, potato, onion, chrysanthemum, eucalyptus, beans, watermelon, corn, strawberry, pepper, rose, soy	not informed	II
Chlorpyrifos	$C_9H_{11}Cl_3NO_3PS$	Organophosphate	Insecticide, ant and acaricide	cotton, potato, coffee, barley, citrus, cabbage, beans, tobacco, apple, corn, grassland, soybean, sorghum, tomato, wheat	not informed	II
Clothianidin	$C_6H_8ClN_5O_2S$	Neonicotinoid	Insecticide	cotton, beans, corn, soybeans	not informed	II
Cyazofamid	$C_{13}H_{13}ClN_4O_2S$	Imidazole	Fungicide	lettuce, potato, broccoli, onion, chinese cauliflower, cauliflower, melon, cabbage, rose, tomato, grape	not informed	U
Chlorantraniliprole	$C_{18}H_{14}BrCl_2N_5O_2$	Anthranilamide	Insecticide	potato, coffee, citrus, melon, soybean, tomato, grape	not informed	U

Dimethoate	$C_5H_{12}NO_3PS_2$	Organophosphate	Insecticide and acaricide	cotton, citrus, apple, tomato, wheat	not informed	II
Dimethomorph	$C_{21}H_{22}ClNO_4$	Morpholine	Fungicide	pumpkin, zucchini, watercress, lettuce, garlic, chilli, potato, onion, spinach, guarana, jiló, papaya, passion fruit, cucumber, pepper, arugula, tomato, grape	3: The agent is not classifiable as to its carcinogenicity to humans	III
Epoxiconazole	$C_{17}H_{13}ClFN_3O$	Triazole	Fungicide	peanuts, coffee, corn, soybeans, wheat	not informed	FM
Famoxadone	$C_{22}H_{18}N_2O_4$	Oxazolidinedione	Fungicide	potato, onion, carrot, citrus, beans, apple, papaya, mango, watermelon, melon, peach, tomato, grape	not informed	U
Fenpyroximate	$C_{24}H_{27}N_3O_4$	Pyrazole	Acaricide	coffee, citrus, coconut, apple, papaya, strawberry, rose, tomato	not informed	II
Fenpropathrin	$C_{22}H_{23}NO_3$	Pyrethroid	Insecticide and acaricide	cotton, coffee, onion, citrus, chrysanthemum, beans, apple, papaya, corn, strawberry, cabbage, rose, soybean, tomato	not informed	II
Fentin	$C_{18}H_{15}Sn$	Organostannic	Fungicide	potato, onion, carrot, cotton, beans, watermelon	not informed	II
Fipronil	$C_{12}H_4Cl_2F_6N_4OS$	Pyrazole	Insecticide, ant and termite	potato, sugar cane and corn	not informed	II
Flubendiamide	$C_{23}H_{22}F_7IN_2O_4S$	Phthalic acid diamide	Insecticide	cotton, corn, soybean, tomato	not informed	III
Flutriafol	$C_{16}H_{13}F_2N_3O$	Triazole	Fungicide	cotton, coffee, sugarcane, beans, corn, soybeans, wheat	not informed	II
Glufosinate	$C_5H_{12}NO_4P$	Substituted homoalanine	Herbicide and growth regulator	lettuce, cotton, banana, potato, coffee, citrus, eucalyptus, apple, corn,	not informed	II

				nectarine, peach, cabbage, wheat, grape		
Glyphosate	$C_3H_8NO_5P$	Substituted Glycine	Herbicide	irrigated rice, sugarcane, coffee, citrus, apple, corn, pastures, soybeans, tobacco, grapes	2A: Probably carcinogenic to humans (IARC 112)	III
Hexythiazox	$C_{17}H_{21}ClN_2O_2S$	Thiazolidinecarboxamide	Acaricide	coffee, coconut, mango	not informed	U
Imazalil	$C_{14}H_{14}Cl_2N_2O$	Imidazole	Fungicide	banana, citrus, apple, papaya, mango, watermelon, melon	2B: The agent is possibly carcinogenic to humans	II
Imidacloprid	$C_9H_{10}ClN_5O_2$	Neonicotinoid	Insecticide	cotton, potato, sugarcane, onion, chrysanthemum, beans, melon, tomato	not informed	II
Lambda-Cyhalothrin	$C_{23}H_{19}ClF_3NO_3$	Pyrethroid	Insecticide	watercress, lettuce, cotton, garlic, leek, peanuts, rice, oats, potatoes, broccoli, coffee, sugar cane, onions, chives, barley, citrus, coriander, cabbage, cauliflower, chrysanthemum, beans, fig, tobacco, sunflower, mango, melon, corn, strawberry, forage palm, pasture, cucumber, pepper, cabbage, rose, soybean, sorghum, tomato, wheat, grape	not informed	II
Lufenuron	$C_{17}H_8Cl_2F_8N_2O_3$	Benzoylurea	Insecticide and acaricide	cotton, oat, potato, coffee, sugar cane, canola, rye, barley, citrus, coconut, eucalyptus, sesame, sunflower, linseed,	not informed	III

				camona, apple, millet, corn, cucumber, peach, cabbage, soy, sorghum, tomatoes, wheat, triticale		
Metalaxyl-M	$C_{15}H_{21}NO_4$	Acylalaninate	Fungicide	peanuts, rice, beans, sunflower, corn, grassland, soybean, sorghum	not informed	II
Methidathion	$C_6H_{11}N_2O_4PS_3$	Organophosphate	Insecticide and acaricide	Cotton, apple	not informed	Ib
Methomyl	$C_5H_{10}N_2O_2S$	Oxime methylcarbamate	Insecticide and acaricide	cotton, potato, corn, soybean, tomato, wheat	not informed	Ib
Permethrin	$C_{21}H_{20}Cl_2O_3$	Pyrethroid	Insecticide and antkiller	cotton, rice, citrus, cabbage, beans, tobacco, corn, cabbage, soybean, tomato, wheat, grape	3: The agent is not classifiable as to its carcinogenicity to humans	II
Pyraclostrobin	$C_{19}H_{18}ClN_3O_4$	Strobilurin	Fungicide	cotton, peanuts, oats, potato, sugarcane, onion, carrot, citrus, chrysanthemum, beans, sunflower, apple, mango, melon, corn, cucumber, pepper, rose, soybean, sorghum, tomato, wheat	not informed	U
Pirimiphos-methyl	$C_{11}H_{20}N_3O_3PS$	Organophosphate	Insecticide and acaricide	rice, barley, citrus, cabbage, corn, wheat	not informed	II
Propargite	$C_{19}H_{26}O_4S$	Alkyl sulfite	Acaricide	cotton	not informed	III
Propiconazole	$C_{15}H_{17}Cl_2N_3O_2$	Triazole	Fungicide	garlic, peanuts, rice, banana, coffee, barley, beans, gladiolus, corn, rubber tree, wheat	not informed	II
Profenofos	$C_{11}H_{15}BrClO_3PS$	Organophosphate	Insecticide and acaricide	cotton, peanuts, potatoes, coffee, onions, peas, beans, beans, watermelon, corn, cucumbers,	not informed	II

				cabbage, soybeans, tomatoes		
Trifloxystrobin	$C_{20}H_{19}F_3N_2O_4$	Strobilurin	Fungicide	coffee, barley, soybean, wheat	not informed	U
Triflumuron	$C_{15}H_{10}ClF_3N_2O_3$	Benzoylurea	Insecticide	cotton, potato, sugarcane, tobacco, corn, soybean, wheat	not informed	U
Trinexapac-ethyl	$C_{13}H_{16}O_5$	Oxocyclohexanecarboxylic acid	Growth regulator	Oat, sugar cane, barley wheat	not informed	III
Thiamethoxam	$C_8H_{10}ClN_5O_3S$	Neonicotinoid	Insecticide	peanuts, rice, potatoes, onions, beans, corn, grassland, cucumbers, soybeans, sorghum, tomatoes, wheat	not informed	II

Legend: Table 4 presents data on the active substances of pesticides with the highest volume of sales in Brazil according to ANVISA (1). Agents Classified by the IARC Monographs, Volumes 1–132: Group 1 = Carcinogenic to humans; Group 2A = Probably carcinogenic to humans; Group 2B = Possibly carcinogenic to humans; Group 3 = Not classifiable as to its carcinogenicity to humans (2). WHO index classification of pesticide active ingredients by Hazard and Guidelines to Classification: Ia = Extremely hazardous; Ib = Highly hazardous; II =Moderately hazardous; III = Slightly hazardous; U = Unlikely to present acute hazard in normal use; FM = Fumigant, not classified; O = Obsolete as pesticide, not classified (3). 1 Agência Nacional de Vigilância Sanitária - Anvisa <https://www.gov.br/anvisa/pt-br> 2 IARC Monographs On The Identification Of Carcinogenic Hazards To Humans <https://monographs.iarc.who.int/agents-classified-by-the-iarc> 3 WHO recommended classification of pesticides by hazard and guidelines to classification, 2019 edition. Geneva: World Health Organization; 2020. Licence: CC BY-NC-SA 3.0 IGO.
